# Microbial and chemical predictors of methane release from a stratified thermokarst permafrost hotspot

**DOI:** 10.3389/fmicb.2025.1657143

**Published:** 2025-10-10

**Authors:** Kevin S. Rozmiarek, Jihoon Yang, Jenna Schambach, Haley Bennett, Tristan A. Caro, Jason Sammon, Joshua J. Whiting, Philip R. Miller, Bryce Ricken, Lisa Bigler, Richard S. Jayne, David Fukuyama, Tyler R. Jones, Chuck R. Smallwood

**Affiliations:** ^1^Institute of Arctic and Alpine Research, University of Colorado Boulder, Boulder, CO, United States; ^2^Department of Geological Sciences, University of Colorado Boulder, Boulder, CO, United States; ^3^Bioresource and Environmental Security, Sandia National Laboratories, Livermore, CA, United States; ^4^Department of Environmental Systems Biology, Sandia National Laboratories, Albuquerque, NM, United States; ^5^Biological and Chemical Sensors, Sandia National Laboratories, Albuquerque, NM, United States; ^6^Applied Systems Analysis and Research, Sandia National Laboratories, Albuquerque, NM, United States

**Keywords:** methane hotspot, thermokarst soils, methanotrophy, microbial VOCs, permafrost thaw, biogeochemical modeling, methane isotopes, carbon cycling

## Abstract

Soils are dynamic interfaces that can act as both sources and sinks of methane (CH₄), yet the microbial processes underlying these fluxes remain poorly constrained in current Earth system models—particularly in thawing permafrost regions. Accurately quantifying subsurface microbial activity and its response to environmental variation is essential for improving predictions of CH₄ emissions under shifting temperature regimes. Here, we explore the potential of volatile organic compounds (VOCs) as early chemical indicators of microbial processes driving CH₄ production within a thermokarst-associated CH₄ hotspot. Field surveys at Big Trail Lake, a young thermokarst feature in central Alaska, identified localized CH₄ emission zones. Anaerobic soil laboratory microcosms from 50, 200, and 400 cm depths were incubated at −4 °C, 5 °C, and 12 °C to simulate freeze–thaw transitions. Methane flux increased markedly with temperature, and microbial community shifts revealed *Methanosarcina* spp. as the dominant methanogen, particularly at 200 cm. VOC profiling showed strong depth- and temperature-dependent patterns, with the 50 cm layer exhibiting the greatest chemical diversity. Notably, 200 cm soils produced VOC signatures overlapping with those from pure *Methanosarcina acetivorans C2A* cultures, supporting the identification of shared metabolites linked to active methanogenesis. Extended 60-day incubations confirmed temperature-sensitive CH₄ production. Carbon isotopic enrichment in CH₄ was unexpectedly strong with warming, and metagenomic detection of ANME-associated markers–including multiheme cytochromes and formate dehydrogenases–supports temperature-sensitive anaerobic oxidation of methane as a significant control on isotopic signatures. Calculated Q₁₀ values for methanogenesis exceeded typical values for boreal soils, highlighting an underappreciated temperature responsiveness of Arctic methanogens. Together, these results demonstrate that VOCs can serve as informative biomarkers of subsurface microbial activation and offer a novel diagnostic tool for detecting early-stage CH₄ hotspot formation. Incorporating such chemically and biologically resolved metrics into process-based models will be critical for improving forecasts of CH₄ release from thawing permafrost landscapes.

## Background

Methane (CH₄) is the second most influential long-lived radiative driver in the atmosphere after carbon dioxide (CO₂), contributing approximately 16% of anthropogenic radiative forcing, or 0.56 W m^−2^ (Forster et al., 2023; Jackson et al., 2024). Recent increases in global CH₄ emission budgets, now estimated at ~61 Tg CH₄ yr.^−1^, have raised concerns over the stability of natural CH₄ sources under increasing environmental variability (Michel et al., 2024; Turetsky et al., 2019). Wetlands, including thawing permafrost zones, are major contributors to atmospheric CH₄, but predicting their emissions remains uncertain due to the complexity of biogeochemical and physical drivers (Zhang et al., 2023; Voigt et al., 2023; Jiao et al., 2025; Yuan et al., 2024). Isotopic analysis, particularly δ^13^C-CH₄, provides a valuable forensic tool for distinguishing CH₄ sources and sinks and tracking carbon flux dynamics (Michel et al., 2024; Basu et al., 2022). However, isotopic source attribution in wetlands remains limited by sparse, and incomplete biogeochemical data. This gap is particularly pronounced in Arctic permafrost systems undergoing thaw, where both microbial and abiotic controls on CH₄ flux remain poorly constrained (Lan et al., 2021). Biologically, CH₄ flux is governed by the balance between methanogenesis and methanotrophy (Guerrero-Cruz et al., 2021). Soils act as both a CH₄ source and sink, with aerobic and anaerobic methanotrophs consuming up to 45 Tg CH₄ yr.^−1^ globally (Saunois et al., 2020; Ellenbogen et al., 2024). Yet, methanotrophic pathways remain elusive, especially in cold, oxygen-limited soils (Jiang et al., 2025). Environmental stressors—such as hydrologic shifts, oxygen gradients, and substrate limitation—can suppress CH₄ uptake while promoting methanogenesis. Characterizing microbial ecology in these systems has been hindered by cultivation challenges and lack of integrative field-scale biogeochemical data.

In these landscapes, permafrost degradation leads to heterogeneous soil conditions—altering redox dynamics, carbon availability, and microbial activity in ways that are difficult to resolve using conventional metrics alone. Conventional measurements such as thaw depth, soil temperature, soil moisture, and bulk carbon content provide essential baseline information but are limited in their ability to capture fine-scale variability or early biogeochemical transitions (Walter Anthony et al., 2024; Liljedahl et al., 2016). These approaches often assume homogeneity across spatial scales and may miss subsurface “hot spots” or “hot moments” of activity. Critically, they do not resolve dynamic microbial responses, spatially variable redox zonation, or transient signals of incipient metabolic activity—such as the production of volatile organic compounds (VOCs) or expression of functional genes involved in anaerobic carbon cycling—that precede detectable CH₄ emissions (Jiao et al., 2023; Ghirardo et al., 2020; Kramshøj et al., 2018; Kramshøj et al., 2019). As a result, emerging molecular, isotopic, and metabolic indicators are increasingly recognized as necessary complements to traditional environmental monitoring, offering earlier and more mechanistic insights into the biogeochemical feedbacks of permafrost thaw (Schuur et al., 2015; Turetsky et al., 2020; Smallwood et al., 2025).

Microbial VOCs, byproducts of both primary and secondary metabolism, represent a promising chemical fingerprint of microbial function and community shifts (Pozzer et al., 2022; Kietäväinen et al., 2025). VOCs diffuse across soil matrices and can reflect metabolic pathways such as fermentation, amino acid catabolism, and anaerobic carbon degradation (Kramshøj et al., 2018; Smallwood et al., 2025; Lemfack et al., 2018; Albers et al., 2018). Because VOCs travel farther than many aqueous-phase metabolites and vary predictably with temperature, oxygen, and substrate, they offer a window into subsurface microbial biokinetics—especially under dynamic thaw conditions (Kramshøj et al., 2019; Insam and Seewald, 2010).

Here, we examine how subsurface microbial community structure and metabolic function shift across a laboratory-simulated seasonal temperature gradient in an actively thawing thermokarst system: Big Trail Lake (BTL) in Goldstream Valley, Alaska. BTL is a young thermokarst lake that formed between 1949 and 1967 due to abrupt permafrost thaw (Walter Anthony et al., 2018). The lake is underlain by a talik that extends to approximately 10–15 m, indicating extensive permafrost degradation (Pellerin et al., 2022). BTL is characterized by high emissions of ^14^C-depleted CH₄, evidence of abrupt permafrost thaw, making it an ideal hotspot for observing dynamic CH₄ cycling (Walter Anthony et al., 2018; Elder et al., 2021). We attribute the lower radiocarbon (^14^C) content of CH₄ in the rapidly thawing talik to the preferential mobilization of older, deeper soil carbon pools. Abrupt thaw processes expose previously frozen organic matter more quickly, leading to methane generation from substrates that have been sequestered longer in the permafrost column. This phenomenon has been previously observed at Big Trail Lake (Walter Anthony et al., 2018), and highlights how thermokarst dynamics, including lateral and vertical thaw, influence carbon age and methane signatures (Table 1).

**Table 1:** Shared KEGG-annotated compounds detected in thermokarst soil incubations and *Methanosarcina acetivorans* cultures.

	Compound	KEGG Compound ID	BTL 50 cm (5 °C)	BTL 50 cm (12 °C)	BTL 50 cm (−4 °C)	BTL 200 cm (5 °C)	BTL 200 cm (12 °C)	BTL 200 cm (−4 °C)	BTL 400 cm (5 °C)	BTL 400 cm (−4 °C)	MaC2A (2 °C)	MaC2A (37 °C)
KEGG compounds overlapping MaC2A and BTL200 cm	Acetophenone	C07113	+	+	+	+	+	−	−	−	+	+
	Benzaldehyde	C00261	+	+	+	+	+	+	+	+	+	+
	Biphenyl	C06588	−	+	−	+	+	−	+	−	+	−
	Butylated Hydroxytoluene	C14693	−	+	+	+	−	+	+	+	−	+
	Decanal	C12307	+	+	−	+	+	−	+	+	+	+
	Dodecanal	C02278	−	+	−	−	−	−	−	+	−	+
	Dodecane	C08374	+	−	−	−	+	−	+	+	+	+
	Hentriacontane	C08376	+	+	+	−	−	+	+	+	−	+
	Methyl Alcohol	C00132	−	−	−	−	−	+	−	+	−	+
	Nonanoic acid	C01601	+	+	+	−	+	−	−	−	−	+
	o-Xylene	C07212	+	+	+	+	+	+	+	+	+	+
	Octanoic acid	C06423	+	+	+	−	+	−	−	−	−	+
	p-Xylene	C06756	−	+	−	−	+	−	−	−	+	+
	Pentanoic acid	C00803	−	+	+	−	+	+	−	+	+	+
	Phenol	C00146	+	+	+	+	+	−	−	+	+	−
	Tridecane	C13834	+	−	−	−	+	−	+	+	+	+

Green shading indicates the presence of each compound under specific incubation conditions across depths (50, 200, and 400 cm) and temperatures (−4 °C, 5 °C, 12 °C), as well as in pure cultures of *M. acetivorans C2A (MaC2A)*. Overlapping metabolites highlight potential VOC biomarkers linking *in situ* microbial activity to methanogen-specific metabolic pathways.

Using a combination of high-resolution gas flux measurements, CH₄ carbon isotope analysis, VOC profiling, and shotgun metagenomics, we evaluated the evolving biogeochemical environment over a two-month period. We hypothesize that VOC fingerprints, in tandem with microbial and isotopic data, can illuminate previously unresolved constraints on CH₄ flux in thermokarst environments. By linking microbial function with geochemical dynamics, we aim to improve mechanistic understanding of subsurface CH₄ cycling and provide process-level insights to inform predictive models of Arctic carbon feedbacks (Table 2).

**Table 2:** VOC classes identified in permafrost soil incubations, categorized by associated microbial processes, putative microbial sources, and characteristic thaw stages.

VOC Class	Associated process	Microbial source	Thaw stage
Organometallics	Cofactor biosynthesis, methanogenesis (Thauer, 2011)	*Methanosarcina* spp.	Deep thaw (12 °C)
Benzenoids	Secondary metabolism, signaling (Nazaries et al., 2013)	Multiple anaerobes	All stages
Organic nitrogen compounds	Cryo-stress response, turnover byproducts (Mackelprang et al., 2011)	General permafrost microbes	Freeze/thaw (−4 °C)
Phenylpropanoids	Lignin degradation, oxidative stress (McGivern et al., 2024)	Facultative anaerobes	Freeze/thaw
Hydrocarbons	Lipid turnover, maintenance respiration (Waldrop et al., 2023)	Core anaerobic microbiome	Persistent (all depths)

Organometallics and benzenoids are linked to methanogenic and signaling functions, respectively, while organic nitrogen compounds and phenylpropanoids reflect stress responses and substrate degradation under freeze–thaw dynamics. Hydrocarbons appear as persistent signals across depths and conditions, potentially serving as baseline indicators of microbial maintenance activity. These classifications support the use of VOC profiles as biomarkers for microbial functional states in thawing permafrost systems.

In thawing permafrost soils, microbial VOCs are byproducts of anaerobic carbon metabolism, fermentation, and stress-related cellular processes. These compounds include short-chain fatty acids (e.g., acetic and propionic acid), alcohols (e.g., ethanol, methanol), ketones (e.g., acetone), aldehydes (e.g., formaldehyde, benzaldehyde), and sulfur-containing volatiles (e.g., dimethyl sulfide) (Rinnan, 2024). Many of these VOCs are associated with fermentative bacteria, methanogenic archaea, and microbial responses to osmotic or oxidative stress. Field studies in Arctic soils have reported VOC concentrations ranging from low nanomolar to low micromolar levels in porewater, with surface fluxes spanning tens to hundreds of nanograms per square meter per hour, depending on temperature, redox conditions, and soil depth (Kramshøj et al., 2019; Insam and Seewald, 2010; Wester-Larsen et al., 2020). These volatiles diffuse more readily than aqueous metabolites, making them valuable early indicators of microbial reactivation during thaw progression.

**Figure 1:**
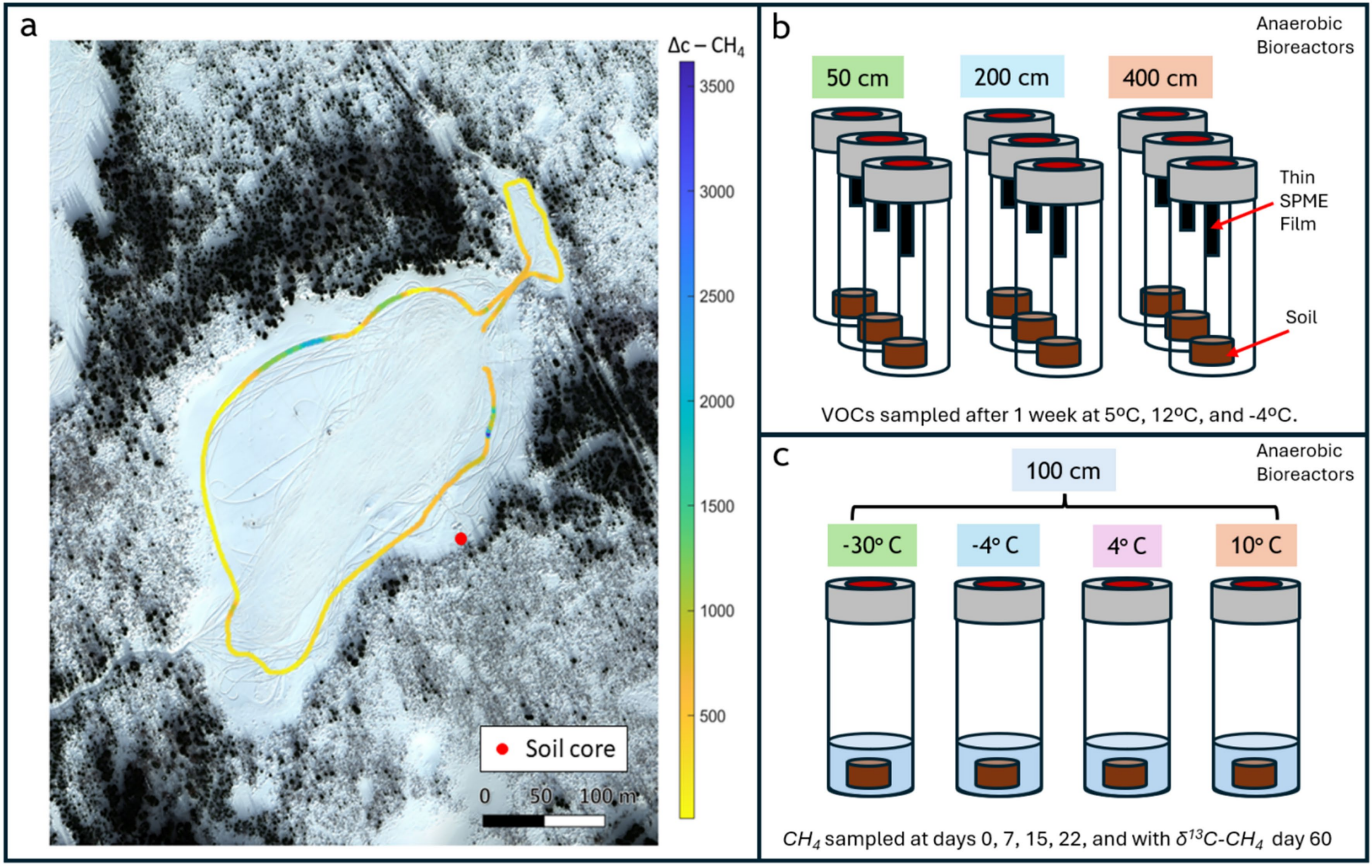
Overview of experimental design for investigating VOC dynamics in thawing permafrost soils. **(a)** Aerial view of the thermokarst lake system in Alaska with outlined sampling transect and soil core collection site. **(b)** Anaerobic microcosm setup for VOC measurement showing triplicate incubations for each depth, housed in bioreactors with VOC-trapping thin films. **(c)** Anaerobic microcosm setup for methane carbon isotope mole fraction measurement across different temperatures at 100 cm depth, housed in bioreactors and slurred with added Milli-Q water.

To improve geochemical characterization of thermokarst thaw features, we subsampled a frozen soil core collected from the BTL field site for use in two controlled incubation experiments (Figure 1). Both experiments were designed to examine the temperature dependence of microbial activity and associated biogeochemical outputs, using temperature as a proxy for thaw progression. Incubation temperatures were chosen to span sub-zero and above-freezing conditions (4 °C, 10 °C, and 20 °C) to capture a broad range of microbial activity potentials relevant to seasonal variation and projected warming. Although continuous in-situ temperature data are not available across the lake margin, the chosen temperatures reflect typical soil thermal regimes near the thaw front during spring through late summer. These conditions also promote detectable metabolic activity, allowing us to assess functional shifts across a representative thermal gradient.

In the first incubation, we focused on the characterization of volatile organic compounds (VOCs), targeting a broad suite of microbial metabolites to assess non-methane geochemical signals potentially linked to methanogenic activity (Figure 1b). In the second incubation we measured CH₄ flux across a temperature gradient, followed by isotopic analysis of CH₄ to determine its carbon-isotopic composition (δ^13^C-CH₄) at the conclusion of the experiment (Figure 1c). Shotgun metagenomic sequencing was performed on samples from both incubations to assess microbial community composition and functional potential as a function of temperature and soil depth. This approach enabled integrated analysis of microbial, chemical, and isotopic indicators of thaw-driven biogeochemical change.

## Methods and materials

### Study site description and methane measurements

Sediment core samples were obtained in Goldstream Valley, Alaska during prior fieldwork at Big Trail Lake site 1 (referred to BTL in this paper) in March 2023 at GPS coordinates 64.91890 N, 147.81996 W as we previously reported in Smallwood et al. (2025). Sampling equipment was sterilized using 10% bleach followed by 70% ethanol or autoclaved prior to sub-sampling. In March 2023, we conducted a CH₄ emission survey using a handheld optical spectrometer (Quanta3) around the littoral boundary of Big Trail Lake (BTL) and a small feeder pond to the north, concurrent with core sampling (Figure 1a).

### Measuring carbon-isotope production rates in soil incubations

Samples from the BTL sediment core were shipped to the Institute of Arctic and Alpine Research (INSTAAR), University of Colorado. Samples were stored in an insulated incubation chamber built at INSTAAR kept at 4 °C to ensure biological fidelity with naturally occurring temperature conditions at the time of sample acquisition. Soil aliquots were separated from the BTL core at 100 cm depth below ground surface. The aliquots were used for incubations targeting temperature-dependent production rates of CH₄ and CO₂ specific to the BTL site. Four ~5 g aliquots (wet mass) were separated from homogenized soil via a surface-sterilized chisel and trowel. Aliquots were placed in a 60 mL jar with 5 mL of Milli-Q water (MilliporeSigma, Burlington, Massachusetts) and homogenized. Jars were then sealed and sparged with nitrogen gas to create an anoxic environment. Samples were then incubated in the dark at temperatures of −30 °C, −4 °C, 4 °C, and 10 °C. Samples were measured at days 0, 7, 15, 22, and 60. It is expected that little microbial activity occurs at −30 °C, increasing starting at −4 °C, and further at 4 °C and 10 °C. At BTL, large step increases in biodiversity of microbial populations have been observed (Smallwood et al., 2025). At all timepoints besides day 60, samples were analyzed for CH₄ and CO₂ concentration vis gas-chromatography flame ionization detection/thermal conductivity detection (GC-FID/TCD). Using a gas-tight syringe, 1 mL samples were injected into a GC-FID/TCD (SRI 8610C). Syringes were filled with 1 mL of nitrogen gas and injected prior to pulling CH₄ to ensure bottles maintained constant pressure. The GC-FID/TCD was calibrated against a 1% CH₄/CO₂ certified reference gas mixture (Scott Specialty Gases, Cat. No. 22561; now part of Air Liquide, Radnor, PA, USA) injected in variable amounts. Error for measurements was determined via the nearest calibration point’s first standard deviation.

To understand carbon isotopic mass balance, soil and headspace carbon-isotopic composition was determined. Soil organic-matter carbon-isotope composition was measured via combustion isotope-ratio mass spectrometry (c-IRMS). Samples were introduced for combustion to a FlashSmart Elemental Analyzer coupled to DELTA Q IRMS (Thermo Fisher Scientific, Waltham, Massachusetts). Carbon-isotope composition was calibrated to Vienna Pee Dee Belemnite (VPDB) via a suite of calibrated secondary references. At day 60, headspace gas was pulled from incubation headspace via a gas-tight syringe, diluted with nitrogen gas in another syringe, and slowly injected into a cavity-ring down spectrometer G2202-i Isotopic Analyzer (Picarro Inc., Santa Cruz, California). Samples were again calibrated to VPDB via a suite of calibrated secondary gas references.

### Soil core subsampling for VOC incubations

Core subsamples were extracted from the BTL core at depths of 50 cm, 200 cm, and 400 cm. At each depth, three replicate samples were collected. Each core subsample was sectioned using a sterile 5 mL syringe with syringe tip excised, then the syringe plunger was used to push the core into individual 15 mL conical tubes for each replicate. To prepare incubation vials, 2 g of soil were weighed and transferred from the conical tube to a labeled 20 mL screw cap amber vials containing a thin-film holder (Gerstel GmbH, Mulheim, Germany). The pH of each triplicate sample was collected by combining 0.5 g of soil in 1 mL of DI water and measured with the Mettler Toledo S220 SevenCompact™ Benchtop pH/ISE Meter (Mettler Toledo, Columbus, Ohio). Values between triplicates were averaged for the pH of each depth. Both the 15 mL conical and incubation vials were stored at −20 °C until the incubation experiment.

### Soil VOC incubation conditions

Soil incubation vials contained 20 mm x 4.65 mm Thin-Film-Solid-Phase-Micro-Extraction (TF-SPME) coated with 90 μm mixture of polydimethylsiloxane (PDMS) and a copolymer of divinylbenzene and vinyl pyrrolidinone (HLB) (Gerstel GmbH, Mulheim, Germany). Before sampling, all TF-SPME’s were conditioned using a TurboMatrix 220 tube conditioner (Perkin-Elmer, Shelton, Connecticut) under inert gas flow at 250 °C for 60 min. The sample vials were incubated for a total of 23 days at varying temperatures with intermittent volatile organic compound (VOC) and microbial sampling. The samples were held at 5 °C for the first 7 days, 12 °C for next 8 days, and −4 °C for the final 8 days. These incubation temperatures approximate a range of thawed and re-frozen conditions expected in permafrost soils across spring and summer seasons, capturing microbial activity across a realistic thermal gradient. Incubation tubes were kept in the Lab Armor® 6 L Bead Bath (Lab Armor, Plano, TX) for the first two temperature intervals, and in a Danby Chest Freezer (Danby, Ontario, CA) for the last. Microbial sampling was performed the day following a change in temperature, and VOCs were sampled every 2–5 days.

### Soil VOC collection

VOC were passively collected on TF-SPMEs in anaerobic bioreactors (Figure 1b). TF-SPME harvest and replacement was performed in a BACTRON600 Anaerobic Chamber (Sheldon Manufacturing, Cornelius, Oregon) containing a VOC scrubber fan to prevent exposure to oxygen and contaminating VOCs. To sample for VOC’s, TF-SPME of the incubation vial were collected and replaced at pre-determined time points. To replace the TF-SPME, the sampled TF-SPME was removed with sterile tweezers and placed into an empty stainless steel thermal desorption unit (TDU) tube (Camsco, Houston, Texas), then immediately sealed with brass compression caps fitted with PTFE ferrules (Camsco, Houston, Texas) to prevent contamination. The TDU tubes containing sampled TF-SPME were stored at −20 °C until GCxGC-TOFMS analysis.

### VOC collection from cultured methanogen

VOC collection and analysis were performed on a methanogen species to find overlap between the pure culture methanogen and the soil samples VOC signatures. A thin film holder (Gerstel GmbH, Mulheim, Germany) was stapled to butyl rubber stopper and all supplies, besides the TF-SPME, were autoclaved before transferring to the anaerobic chamber. A fresh stock culture was created from an existing culture of *Methanosarcina acetivorans* strain C2A in late exponential phase by taking 0.5 mL existing culture and measuring optical density at 600 nm on the Thermo Scientific™ Invitrogen™ Nanodrop™ One Spectrophotometer with WiFi and Qubit™ 4 Fluorometer (Thermo Fisher Scientific, Waltham, Massachusetts). The *M. acetivorans* culture was then diluted to an OD of 0.2 in a high salt medium with 50% methanol added at 0.5% of the total culture volume. Using a serological pipette, 5 mL *M. acetivorans* stock culture was dispensed into 12 hungate tubes (Chemglass Life Sciences, Vineland, NJ); 6 of which were autoclaved. 5 mL of a defined high-salt (HS) media (Benedict et al., 2012) with 0.5% methanol was dispensed into an empty tube (media blank). Three culture tubes of each treatment (non-autoclaved and autoclaved; 6 total) and media blank were fitted with a TF-SPME for VOC capture and subsequently left undisturbed for the duration of the experiment. The remaining cultures were used to monitor growth, so optical density was measured daily by extracting 300 μL *M. acetivorans* culture with sterile 1 mL syringe and 23G needle, then combining with 300 μL HS medium in semi-microcuvette. All 13 samples were incubated in a biobag held in 37 °C anaerobic chamber incubator. After 12 days of incubation, each TF-SPME was removed with sterile tweezers and placed into TDU tubes that were then stored in 4 °C until GCxGC-TOFMS analysis.

### Microbial sampling

Following a change in incubation temperature, soil was sampled to determine the microbial community via metagenesis. In the anaerobic chamber, a food-grade plastic straw (Up&Up, Brooklyn, New York) was inserted into the sample to extract ~0.5 g. The straw containing soil was cut and inserted into a sterile 2 mL microcentrifuge tube. The microcentrifuge tubes were stored at −20 °C until sent for sequencing.

### Shotgun metagenomic library preparation and sequencing

For shotgun metagenomics, DNA was extracted with ZymoBIOMICS®-96 MagBead DNA Kits (Zymo Research, Irvine, CA). Extracted genomic DNA was subjected to shotgun metagenomic sequencing. The Illumina DNA Prep Kit (Illumina, San Diego, CA) was used to prep sequencing libraries with ~100 ng DNA. All libraries were quantified with TapeStation® (Agilent Technologies, Santa Clara, CA) and then pooled to equal abundance. The final pool was quantified using qPCR. All metagenomic libraries were sequenced on the Illumina NovaSeq® X platform (2 × 150 bp paired-end configuration). All samples were pooled and sequenced in a single run, eliminating concerns about platform-specific batch effects. No batch correction was required, as library preparation and sequencing were performed under consistent conditions.

### Metagenomic bioinformatics and microbial composition analysis

To remove low quality features raw sequence reads were trimmed with Trimmomatic-0.33 (Sihi et al., 2019); quality trimming by sliding window with 6 bp window size and a quality cutoff of 20 and reads with size lower than 70 bp were removed. Host-derived reads were removed with Kraken2 and sdust was used to detect and remove low-diversity reads (Wood et al., 2019). The DIAMOND sequencer aligner genetically identified antimicrobial resistance and virulence factor against NCBI reference databases (Buchfink et al., 2014). Bacteria and archaea were identified using the GTDB species representative database (RS207). Sourmash (Brown and Irber, 2016) profiled microbial composition, and GenBank databases, also provided by Sourmash, were used to identify virus, protozoa, and fungi. Using BWA-MEM (Li et al., 2024), reads were mapped back to the genomes identified by Sourmash, and microbial abundance was determined based on the count of map reads. The resulting taxonomy and abundance information were further analyzed: (1) alpha- and beta-diversity analyses (Supplementary Figure 2); (2) microbial composition bar plots using QIIME (Morris et al., 2013); (3) abundance heatmaps with hierarchical clustering (based on Bray–Curtis dissimilarity); and (4) biomarker discovery with LEfSe (Monod, 1949) with default settings (*p* > 0.05 and LDA effect size >2). Functional profiling was also performed on assembled reads using EggNOG Mapper (v2.1.12) on genes called from the assembled reads by Prodigal (v 2.6.2) to identify the presence of KOs (KEGG Orthology groups) (Kanehisa et al., 2023; Cantalapiedra et al., 2021).

To evaluate patterns in microbial community composition across environmental gradients, we performed Principal Component Analysis (PCA) on standardized genus-level relative abundance data. Abundances were first log-transformed (if applicable) and then scaled using z-score normalization. PCA was conducted using the scikit-bio package in Python (v0.6.3), and the first two principal components were visualized to assess sample clustering by depth and incubation temperature (Rideout et al., 2023). Sample metadata (depth and temperature) was extracted from column names to facilitate grouping and interpretation.

### GCxGC-TOFMS instrumental parameters and standards

The VOC samples were thermally desorbed using a Gerstel Thermal Desorption Unit 3.5 + (Gerstel GmbH, Mülheim, Germany) integrated into a LECO Pegasus BT4D comprehensive two-dimensional gas chromatograph with time-of-flight mass spectrometer (GCxGC-TOFMS) system (LECO, St. Joseph, MI) and ramped at 60 °C/min from 35 °C to 250 °C and held for 5 min (Smallwood et al., 2025). Desorbed samples were refocused on a Gerstel CIS 4 Cryogenic Inlet, held at −50 °C during TDU desorption and ramped at 12 °C/s to 300 °C to inject the desorbed sample as a single bolus into the GCxGC-TOFMS equipped with a 15 meter, 0.25 mm ID DB-WAX primary column and 2 meter, 0.25 mm ID DB-1 secondary column (Both Agilent, California, United States). The GCxGC-TOFMS system had an initial temperature of 35 °C, ramping at 10 °C/min to a maximum of 230 °C with a 5-min hold. The secondary column was ramped at the same rate, but with a + 5 °C offset from the primary column. The system uses a LECO quad jet thermal modulator design operated with liquid nitrogen cooled nitrogen as the cold jet and heated nitrogen as the hot jet. The modulation period was 4 s, using liquid nitrogen-cooled nitrogen for the cold jet and heated nitrogen for the hot jet. Mass spectra were collected at a rate of 200 spectra/s from 20 mu to 550mu at -70 eV. Both the transfer line and ion source temperatures were set to 250 °C, with a detector voltage offset of -30 V.

### Volatile compound composition and abundance analysis

Top-down quantitative analysis of metabolites in the hit lists was performed using ChromaTOF TILE software (v1.2.6) (LECO Corp. Michigan, United States). Each individual VOC feature identified from the TILES analysis was compared to the NIH PubChem database (https://cactus.nci.nih.gov/chemical/structureaccessedJune2024) to check for alternative chemical synonyms. Using a custom script, the TILE feature table for BTL, containing unidentified and identified VOCs, was further processed by ClassyFire, an automated tool for taxonomic classification of chemicals (Djoumbou Feunang et al., 2016). Using the chemical classification appended TILE feature table, each taxonomic classification was binned to the category [i.e., 200 cm (5 °C)] with the highest average peak area for that individual feature. For handling of the pure culture TILE feature table, the average instrument blank and media blank signal were subtracted from the averaged peak areas of both the autoclaved and non-autoclaved cultures to isolate the pure culture signal. Once this analysis was performed on the autoclaved and non-autoclaved cultures, the signals from the autoclaved sample were subtracted from the living sample. In the analysis, any rows containing zero were removed. A heat map was created using all remaining rows for non-autoclaved (37 °C), autoclaved (37 °C), and dormant (2 °C) samples using the mean retention time for dimension 1 (RT1) and mean retention time for dimension 2 (RT2) as row labels.

### Process-based numerical modeling of methane lags during permafrost thaw

To simulate subsurface CH₄ dynamics during permafrost thaw, we used the PFLOTRAN reactive transport simulator with enhancements to the HYDRATE mode originally developed for marine systems (Hammond et al., 2014). CH₄ was treated as a trace gas in a sequentially coupled flow and reactive transport model solving conservation equations for energy, water, and air components. Mass and energy balances accounted for phase interactions, diffusion, Darcy flow, heat transfer, and phase change. Microbial processes—including aerobic respiration, anaerobic methanogenesis, and CH₄ oxidation—were modeled using temperature-dependent Monod kinetics (Monod, 1949). These simulations were further refined using the Dual Arrhenius Michaelis–Menten Greenhouse Gas (DAMM-GHG) framework to capture temperature-sensitive microbial competition and inhibition effects, following the formulation of Sihi et al. (2019). While the underlying reaction kinetics are consistent with Sihi et al. (2019) model parameters were adapted from Malinverno (2010), which describes CH₄ generation and transport in marine sediments saturated with microbially derived gas. The model domain was one-dimensional (1 m × 1 m × 5 m) with 1 cm vertical resolution. Simulations began with a 130-day spin-up to equilibrate temperature, water content, and CH₄ concentrations to boundary conditions derived from the NOAA-CIRES 20th Century Reanalysis (V2) dataset, which spans 1891 to 2011. The temperature boundary condition used for this simulation corresponds to the year 2011. Following spin-up, we simulated CH₄ transport and fluxes over a 60-day summer period under multiple surface temperature scenarios (0 °C to 4 °C) to evaluate CH₄ production and its delayed emergence at the ground surface. Model results were benchmarked against CH₄ fluxes observed in laboratory incubations of BTL 100 cm depth soils, allowing evaluation of subsurface production and transport lags under warming conditions.

## Results

### Methane emission survey of the littoral region around big trail lake

Elevated CH₄ emissions were observed during a March 2023 survey of the littoral boundary of Big Trail Lake (BTL) and an adjacent feeder pond to the north (Figure 1a). CH₄ mole fractions reached up to 12 ppm at 10 cm above ground level (background ~2 ppm), with spatially heterogeneous fluxes evident across the site. Regions highlighted in blue and dark yellow in Figure 1a correspond to high-emission zones, with vertical concentration differences of up to 3,500 ppb between 10 cm and 1.5 m aboveground—indicative of strong surface emissions. The highest CH₄ levels were recorded near the southeast margin of the lake, coinciding with a sediment core collected for permafrost thaw analysis (Figure 1a, red dot). These results confirmed the presence of localized CH₄ hotspots in both the northern and southern littoral zones, reinforcing prior observations of sustained CH₄ enrichment during both cold and growing seasons at BTL.

### Microbial shifts with temperature in soil incubations

**Figure 2:**
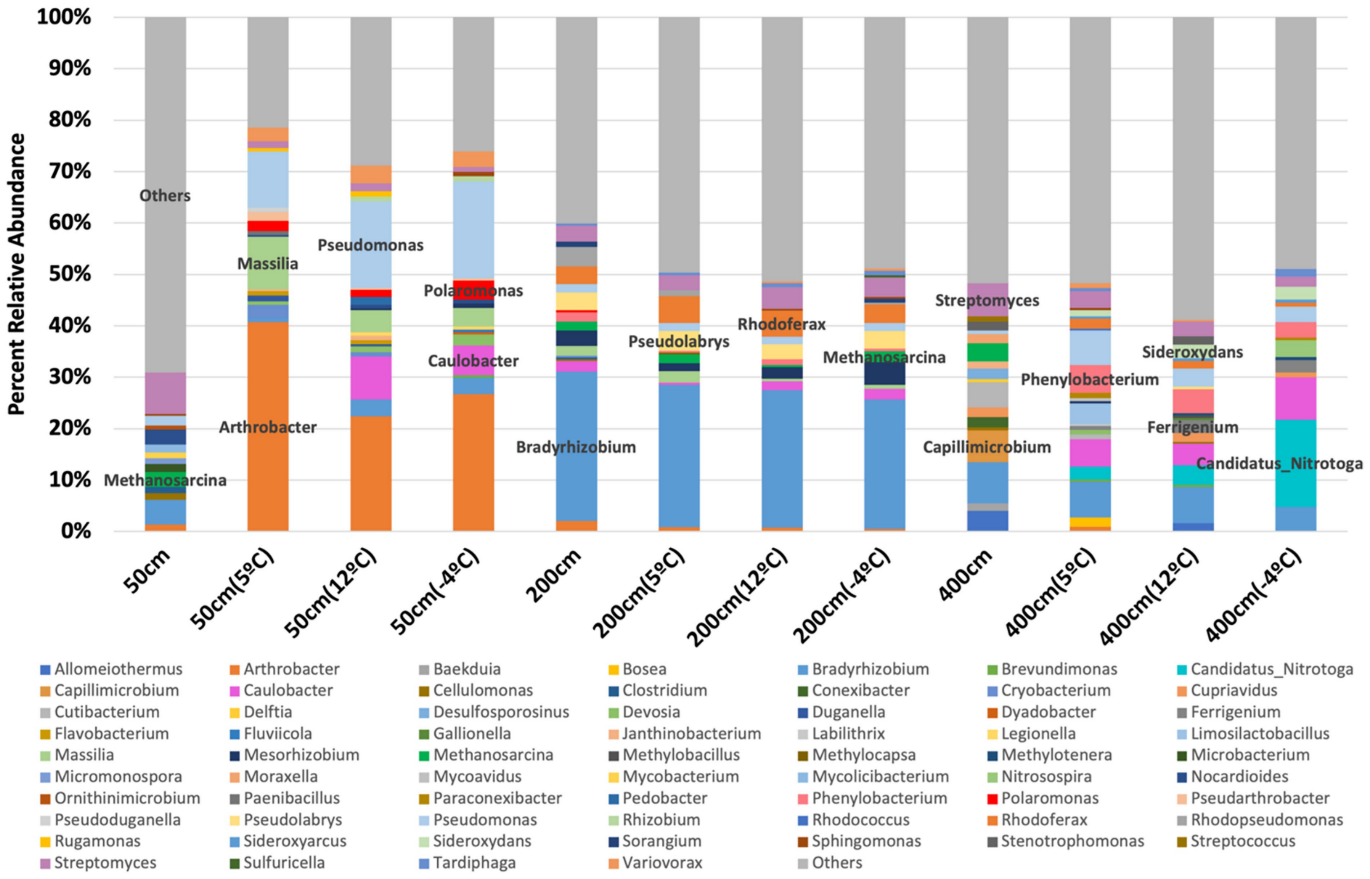
Genus-level microbial community composition in thermokarst soil from Big Trail Lake (BTL) across depth and incubation temperature, based on shotgun metagenomic sequencing. Bar plots represent the average relative abundance of annotated genera from triplicate samples at each depth (50 cm, 200 cm, 400 cm) and temperature condition (−5 °C, 4 °C, and 12 °C). Prominent genera are labeled, and “Others” denotes the combined abundance of taxa contributing <2% relative abundance each. Depth- and temperature-driven shifts are evident, including increased representation of methanogens (e.g., Methanosarcina*), iron-cycling bacteria (e.g.,* Sideroxydans*, Gallionella), and diverse heterotrophs, indicating microbial reorganization during simulated thaw*.

**Figure 3:**
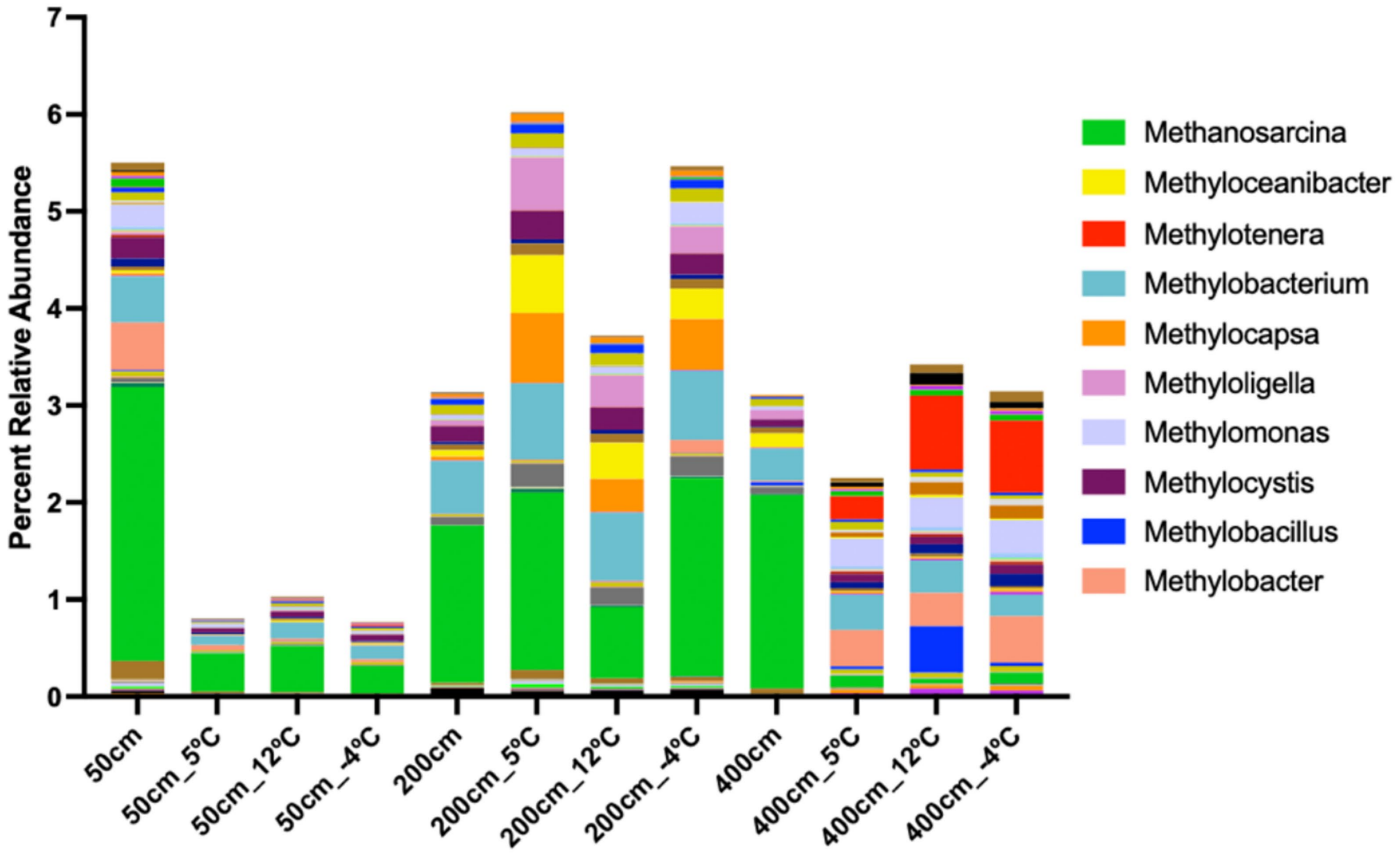
Genus-level distribution of methane-cycling microbial communities across soil depths and incubation temperatures. **S**tacked bar plot showing the relative abundance of methanogenic and methanotrophic genera in anaerobic incubations of thermokarst lake soil from 50 cm, 200 cm, and 400 cm depths, incubated at 5 °C, 12 °C, and −4 °C. Methanosarcina (green) dominates across all depths, with pronounced enrichment at 200 cm under warming (12 °C) and freeze–thaw (−4 °C) conditions, suggesting elevated methanogenic potential. Methylotrophic genera such as *Methylobacterium*, *Methylotenera*, and *Methylocystis* also show depth- and temperature-specific shifts, reflecting dynamic microbial responses to thaw and freeze cycles. Relative abundance data presented here may be influenced by relic DNA, particularly in permafrost soils. As such, these patterns should be interpreted as potential shifts in community composition, not direct measures of viable cell abundance.

To assess microbial community dynamics with depth and temperature, we incubated BTL soil core subsamples in anaerobic micro-bioreactors (Figure 1b). Incubation temperatures progressed from frozen conditions to 5 °C, 12 °C, and −4 °C in one-week intervals. Metagenomic sequencing was conducted at each interval, alongside GC × GC–MS to analyze volatile organic compound (VOC) profiles. All replicates exhibited similar alpha and beta diversity initially, with the exception of replicate 3 from the 400 cm sample, aligning with its lower alpha diversity (Supplementary Figure 2). Microbial composition shifted following temperature increases from −20 °C to 5 °C, stabilizing at 12 °C, with some community restructuring observed at −4 °C (Figure 2). At 50 cm, *Arthrobacter* dominated, followed by *Pseudomonas*, *Massilia*, and *Caulobacter*. Deeper samples (>50 cm) showed expected transitions toward facultative and obligate anaerobes. For instance, *Bradyrhizobium* (facultative anaerobe) was more prevalent at 200 cm, while *Arthrobacter* (obligate aerobe) declined. The 400 cm samples showed the highest microbial diversity, with notable enrichment of *Candidatus Nitrotoga* at −4 °C—indicative of active nitrogen cycling under cooler conditions. *Methanosarcina*, a key methanogen, was found across depths but showed differential responses to temperature. At 50 cm, its abundance dropped from 2.8 to 0.4% after 5 °C incubation. At 200 cm, it increased slightly at 5 °C, then declined sharply at 12 °C, but rose again to 2% at −4 °C. At 400 cm, *Methanosarcina* abundance was initially high (~2%) but declined rapidly with warming and remained low even at −4 °C. Deeper soils harbored syntrophic communities supportive of methanogenesis, including *Candidatus Nitrotoga*, *Bradyrhizobium*, *Phenylobacterium*, and *Limosilactobacillus*. Stacked bar plots of gene abundance confirmed that *Methanosarcina* was the most abundant CH₄-cycling taxon, particularly at 200 cm (Figure 3). Methanotrophs also increased in relative abundance, especially in 200 and 400 cm soils following incubation at 5 °C, with methanotrophic diversity rising over time.

Principal Component Analysis revealed clear separation of microbial communities based on both sediment depth and incubation temperature (Supplementary Figure 3). The first two principal components accounted for a substantial portion of the variance in the dataset, with samples clustering distinctly by depth (50 cm, 200 cm, and 400 cm) and further stratified by temperature treatments (−20 °C to 12 °C). Shallow samples incubated at higher temperatures tended to group separately from deeper or colder samples, indicating that both environmental variables strongly influence community composition.

### Temperature- and depth-dependent shifts in methane-cycling taxa

Anaerobic incubations of sediment samples from 50 cm, 200 cm, and 400 cm depths revealed distinct shifts in methanogenic and methanotrophic taxa across a stepped temperature regime (Figure 3). Samples were incubated sequentially at 0 °C, 5 °C, 12 °C, and −4 °C, each for one week. At 50 cm, *Methanobacterium* and *Methanosarcina* were the dominant methanogens at 0 °C and 5 °C, indicating adaptation to cold, near-surface conditions (Figure 3). A notable increase in *Methanosaeta* and *Methanoregula* was observed at 12 °C, suggesting these taxa are more responsive to moderate warming. Upon cooling to −4 °C, the overall abundance of methanogens declined, although *Methanobacterium* remained detectable, indicating some cold tolerance. At 200 cm, methanogenic diversity increased with temperature. *Methanoculleus* and *Methanoregula* were particularly enriched at 12 °C but were less abundant at 0 °C and nearly absent at −4 °C. These patterns suggest a greater metabolic activation of methanogens at intermediate depths under warming. At 400 cm, the methanogenic community showed the greatest richness at 12 °C, with *Methanoculleus*, *Methanobacterium*, and *Methanosarcina* as dominant taxa. These groups persisted, though at reduced abundance, during the return to −4 °C, suggesting a more metabolically flexible or resilient methanogen community at depth.

Methanotrophs were most abundant at 50 cm and primarily active at lower temperatures (Figure 3). Type I methanotrophs, including *Methylobacter* and *Methylomonas*, were enriched at 0 °C and 5 °C but declined sharply by 12 °C. At −4 °C, methanotrophic taxa were nearly undetectable. Type II methanotrophs (*Methylocystis*) were observed sporadically at 200 cm, with peak presence at 5 °C; however, they did not persist at other temperatures. At 400 cm, methanotrophs were largely absent across all temperature treatments, indicating that CH₄ oxidation activity is limited in deeper anaerobic sediments.

Methanogenic taxa increased in diversity and abundance with depth and temperature, peaking at 12 °C, particularly in sediments from 200 cm and 400 cm. In contrast, methanotrophs were confined to shallow sediments and favored lower temperatures, with peak abundance at 0 °C and 5 °C in the 50 cm horizon. Cooling to −4 °C led to a general decline in both methanogen and methanotroph abundance, though several methanogenic taxa persisted, particularly in deeper samples. These results indicate a stratified and temperature-responsive community, with deeper sediments favoring methanogenic activity under warming and shallow sediments supporting CH₄ oxidation under cold, anaerobic conditions.

### Temperature-dependent methane flux from long-term incubations

**Figure 4:**
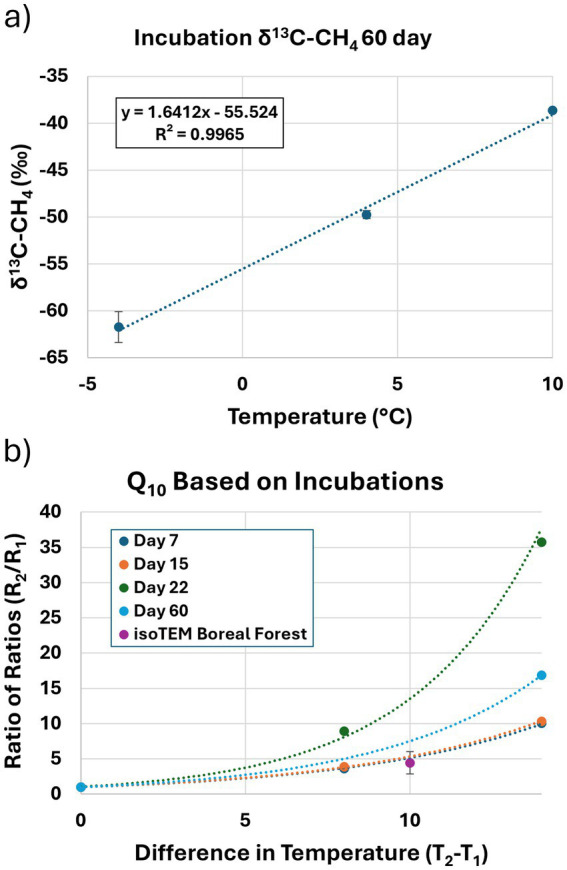
Mesocosm methane incubation results. **(a)** Methane carbon-isotope composition as a function of incubation temperature. Methane carbon-isotopes enrich linearly with response to temperature at the end of a 60-day anaerobic incubation. Error bars report first standard deviation of calibration uncertainty at injected methane concentration. **(b)** Temperature sensitivity of CH₄ flux from thermokarst soil incubations expressed as Q₁₀ values over time. Curves represent the ratio of fluxes (R₂/R₁) across temperature differentials (T₂–T₁) for incubation days 7 (dark blue), 15 (orange), 22 (green), and 60 (light blue). The isoTEM Boreal Forest baseline (purple) represents the average Q₁₀ value typically used in ecosystem models for boreal regions. Lower Q₁₀ values observed at Day 60 indicate elevated methanotrophic communities.

Methane and CO₂ production were tracked over 60-day incubations across temperature treatments (Figure 4; Supplementary Figure 1). Methane accumulation (log-scale) was highest at 10 °C, whereas emissions at −4 °C plateaued between days 22 and 60 (Supplementary Figure 1). Methane fluxes, calculated from CH₄ mass, showed a strong positive correlation with temperature, while CO₂ emissions were less variable across treatments. A transient CO₂ spike during the initial 7 days was attributed to residual oxygen, which diminished by day 15, after which CO₂ production steadily increased—except in −4 °C and −30 °C treatments.

The temperature sensitivity of CH₄ production was quantified using Q₁₀ coefficients. Q₁₀ values were ~5 at days 7 and 15 but rose to 14 by day 22, suggesting that microbial responses to temperature intensify over time (Figure 4b). The average Q₁₀ across all time points was 7.9, considerably higher than values used in many boreal wetland models. Carbon isotope measurements supported these findings (Figure 4a). Soil organic matter had δ^13^C values consistent with C_3_ vegetation (−25.60 ± 0.70‰). Methane δ^13^C values after 60 days ranged from −64.6‰ to −38.9‰ from −4 °C to 10 °C, showing a highly linear response to temperature (R^2^ = 0.997; Figure 4a). Methane was undetectable in −30 °C treatments at day 60 due to low production.

### Temperature-driven functional shifts in microbial communities during 60-day anaerobic incubation

Metagenomic profiles revealed temperature-dependent changes in the relative abundance of key microbial groups with functional roles in fermentation, metal reduction, methanogenesis, and CH₄ oxidation, suggesting possible shifts in metabolic potential across thermal regimes (Supplementary Figure 4). Community composition at the endpoint of the 60-day mesoscale incubation under four temperature treatments (−30 °C, −4 °C, 4 °C, and 10 °C) reflected these trends, with specific groups showing distinct responses to thermal inputs. Anaerobic fermenters such as *Clostridium* were highly abundant under freezing conditions (2.8% at −30 °C) but declined significantly with warming. Other well-characterized fermenters such as *Bacteroides* were consistently present across treatments and may play a central role in sustaining anaerobic carbon turnover. In contrast, less abundant taxa such as *Citrifermentans* and *Paludibacter* exhibited increases at 4 °C (0.98 and 0.23%, respectively). Although these groups were low in relative abundance, they are functionally specialized anaerobes known to contribute to fermentative degradation of organic matter (Thapa et al., 2025; Qiu et al., 2014). Their increased representation at moderate warming suggests a transition in fermentative community composition and highlights the potential ecological relevance of functionally responsive, yet numerically minor taxa. Metal-reducing bacteria (e.g., *Geobacter*, *Geomonas*, and *Geotalea*) were also enriched at 4 °C, with *Geomonas* reaching 2.23% and *Geobacter* peaking at 0.80% (ANOVA, *p* < 0.01), indicating stimulation of dissimilatory metal reduction pathways that could support downstream syntrophic methanogenesis.

Methanogenic archaea also showed temperature-dependent shifts. *Methanosarcina* abundance tripled from 0.44% at −30 °C to 1.27% at 10 °C (ANOVA, *p* < 0.05), while more specialized taxa like *Methanoregula* and *Methanothrix* declined. In parallel, methanotroph-like populations such as *Rhodoferax* and *Polaromonas* also responded strongly to warming. *Rhodoferax* peaked at 6.45% at 4 °C, while *Polaromonas*, typically considered psychrotolerant, rose steadily with temperature from 0.15 to 0.64%, further contributing to potential CH₄ oxidation capacity in warmer conditions. Importantly, two key CH₄ oxidation genes—*xoxF* and *mmoX*—were detected in the metagenomic data with clear temperature-associated trends (R01146 and R01142, respectively in Supplementary Table 2). Both genes were enriched at 4 °C and 10 °C, indicating increased genetic potential for CH₄ and methanol oxidation under warming conditions.

Contigs annotated with these CH₄ oxidation genes were taxonomically linked to non-canonical but increasingly abundant microbial genera, including *Amycolatopsis*, *Geobacter*, *Citrifermentans*, and *Geomonas*. These genera not only increased in abundance with temperature but also carried functional markers (*xoxF*, *mmoX*) associated with CH₄ oxidation, reinforcing the notion that CH₄ oxidation potential increased with temperature and involved a wider phylogenetic range than canonical aerobic methanotrophs.

### ANME marker gene abundance across temperatures during 60-day anaerobic incubation

We quantified the relative abundance of functional marker genes associated with anaerobic methane-oxidizing archaea (ANMEs), including multiheme c-type cytochromes (e.g., *mtrA–H*, *omcX*, *omcI*), hydrogenase complexes (*echA–F*), formate dehydrogenases (*fdhA–F*), and Rnf complex genes (*rnfA–H*) (Supplementary Table 3) (Ouboter et al., 2024). Across the four temperature conditions (−30 °C, −4 °C, 4 °C, 10 °C), the majority of these genes exhibited a distinct temperature-dependent pattern. Gene counts for *mtrC*, *omcI*, *echA*, *fdhA*, *fdhD*, and *rnfC* were consistently highest at 4 °C, with several showing multi-fold increases relative to colder or warmer treatments. For instance, *fdhA* increased from 27 hits at −30 °C to 114 at 4 °C before declining to 60 at 10 °C. Similarly, *mtrC* rose from 8 at −30 °C to 31 at 4 °C. In total, more than 30 ANME-associated genes peaked in abundance at 4 °C, while fewer genes showed maximal counts at −30 °C or 10 °C.

### VOC profiles across depth and temperature

**Figure 5:**
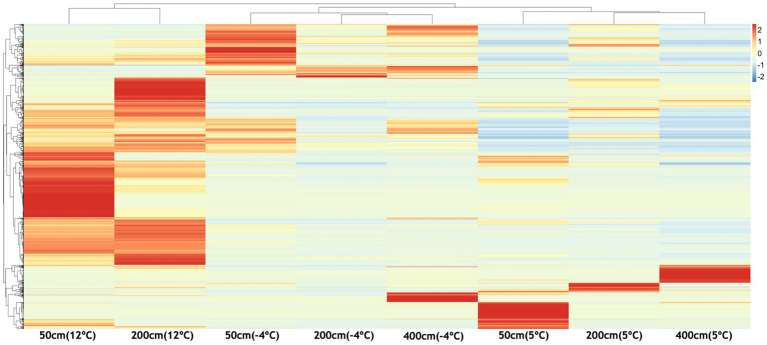
Heatmap showing temperature-dependent differential expression of volatile organic compounds (VOCs) across soil depths (50 cm, 200 cm, 400 cm). VOC intensities were standardized and clustered to reveal patterns of chemical response under warming (5 °C, 12 °C) and re-freezing (−4 °C) conditions. Warmer colors indicate higher relative abundance of specific VOCs. Distinct enrichment patterns at 50 cm and 200 cm under thawed conditions highlight depth- and temperature-specific chemical signatures associated with microbial activity and potential methane hotspot emergence.

**Figure 6:**
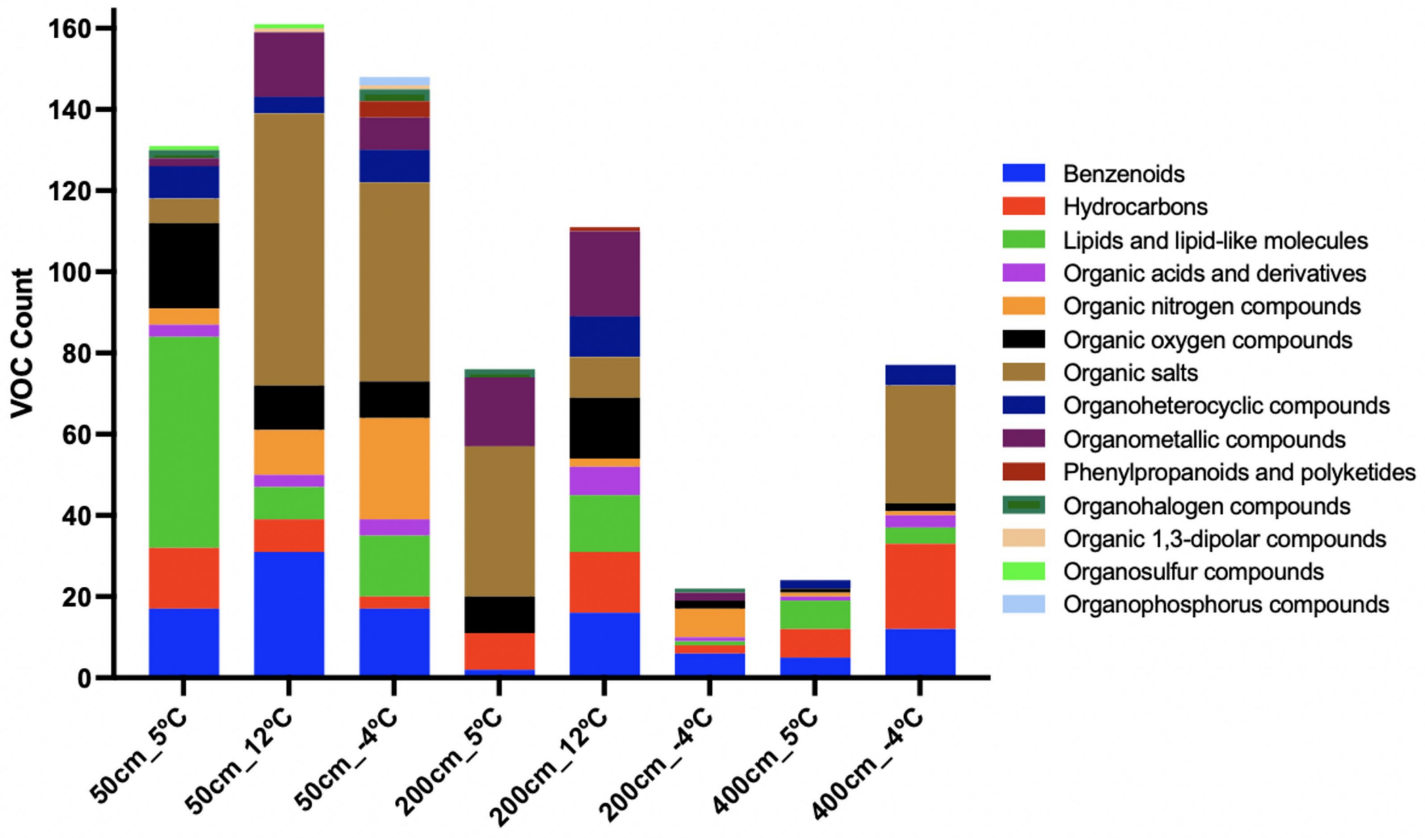
Chemical classification of volatile organic compounds (VOCs) across incubation conditions and temperatures. Bar plots represent the number of VOCs detected per superclass under each experimental condition (50, 200, 400 mg soil or methanogen inoculum; 12 °C, 5 °C, or freeze–thaw “m4C” treatments). High temperatures (12 °C) yielded greater diversity in organometallics, benzenoids, and lipids, particularly in methanogen-enriched incubations (200_12C). Cold or frozen conditions (e.g., 200_m4C, 50_m4C) showed shifts toward organic nitrogen compounds, salts, and stress-associated metabolites such as phenylpropanoids. Minimal background VOCs were observed in blank controls. These profiles suggest distinct VOC fingerprints related to microbial community structure, temperature response, and methanogenic potential.

We detected 2,846 unique VOCs across all incubated samples (Figure 5) during the 1-week temperature steps. Despite all samples producing VOCs, their expression patterns differed significantly by temperature and depth. Samples incubated at 12 °C exhibited the greatest number of highly expressed VOCs across all depths. Unexpectedly, VOC abundances at −4 °C exceeded that at 5 °C in some cases (e.g., 50 and 400 cm), suggesting non-linear temperature dependencies. Hierarchical clustering of VOC profiles revealed consistent temperature-driven grouping. At 200 cm, 2,418 unique VOCs were identified across temperature treatments. A compositional shift in compound classes—particularly between 5 °C and −4 °C—was evident (Figure 6). Organic salts dominated at 12 °C, with organometallics, organic oxygen compounds, lipids, hydrocarbons, and benzenoids also abundant.

The heatmap comparison of VOCs in Figure 5 reveals clear depth- and temperature-dependent patterns in VOC expression across the permafrost soil profiles. At 50 cm depth, the thawing treatments (5 °C and 12 °C) show marked enrichment of specific VOC features, with several compounds showing elevated production and release at 12 °C, indicating strong microbial or chemical activity in the more active surface layer during warming. Upon re-freezing (−4 °C), a subset of these compounds remains elevated, suggesting a legacy effect or persistent volatile production even under cold conditions. At 200 cm, VOC expression at 12 °C also shows moderate enrichment, although the signal is less pronounced than at 50 cm, likely reflecting slower metabolic responses in deeper, less biologically active soil layers.

Notably, some VOCs become more prominent only after re-freezing at this depth, suggesting possible chemical transformation or stress-induced release. The 400 cm layer exhibits relatively low VOC diversity and intensity overall, with limited response to warming. However, several compounds increase specifically under −4 °C conditions, suggesting cryoactive processes or latent metabolic shifts triggered by rapid freeze. It is worth considering whether the low VOC abundance observed at this depth reflects an intrinsically less active microbial community or simply the limited duration of warming applied in these experiments. With sustained warming, deeper communities may have the potential to proliferate and increase VOC production over time, leading to compositional changes. Overall, the data demonstrate that temperature shifts strongly influence VOC dynamics, particularly in shallower soils. These volatile signatures may serve as early indicators of biogeochemical reactivation and microbial community shifts in thawing permafrost, with potential implications for forecasting CH₄ flux and monitoring permafrost-affected ecosystems.

### Temperature- and depth-dependent classification of VOCs

Chemical classification of VOCs revealed distinct patterns across temperature treatments and soil depths, reflecting both thermal sensitivity and microbial source specificity in metabolite production (Figure 6). The distribution of chemical super classes highlights the functional stratification of microbial metabolism and stress responses in thawing permafrost environments.

At 200 cm and 12 °C VOC profiles were enriched in organometallic compounds, benzenoids, organic oxygen compounds, and hydrocarbons. Lipids and lipid-like molecules, along with organoheterocyclic compounds, were also abundant (Figure 6). This chemically diverse profile is consistent with active methanogenic metabolism and secondary metabolite production, suggesting organometallic VOCs as potential biosignatures of archaeal metabolic activity. At 5 °C, the VOC profile at 200 cm shifted toward a less diverse set, dominated by organic salts and organometallic compounds, indicative of reduced but ongoing microbial metabolism. At −4 °C, a further shift occurred, with organic nitrogen compounds and benzenoids becoming prominent. These changes suggest the emergence of stress-induced metabolites, likely associated with cryo-preservation mechanisms or slowed anabolism. VOC diversity was highest in surface soil incubations. At 12 °C, the 50 cm profile was also dominated by organic salts, benzenoids, and organometallic compounds, along with substantial contributions from organic nitrogen and oxygen compounds. These signatures suggest broad microbial activation. At 5 °C and 50 cm depth, lipids and lipid-like molecules were the dominant class, followed by organic oxygen compounds and benzenoids, pointing to membrane turnover and biosynthesis under moderate cold stress. Re-freezing at −4 °C shifted the VOC profile again, with organic nitrogen compounds and organic salts becoming most abundant. Notably, phenylpropanoids and polyketides appeared uniquely under this condition, implying potential roles in microbial stress defense or signaling. In the deeper 400 cm samples, overall VOC diversity was lower. At −4 °C and 400 cm depth, hydrocarbons and organic salts were the dominant classes, possibly linked to cryoactive processes or microbial persistence. At 5 °C and 400 cm, lipids and benzenoids were present, indicating minimal metabolic activity, potentially tied to membrane remodeling or low-level aromatic degradation. VOC presence in instrument and vial blanks was minimal, limited primarily to lipids, organic oxygen compounds, and benzenoids. This low background confirms the biological origin of VOC signals observed in experimental treatments.

### Overlap of VOCs between BTL soils and *Methanosarcina acetivorans*

Sixteen KEGG-annotated volatile organic compounds (VOCs) were identified as overlapping between BTL soil incubations and pure cultures of *M. acetivorans* (Supplementary Figure 5; Table 1). Columns represent experimental conditions for BTL soils at 50 cm, 200 cm, and 400 cm incubated at −4 °C, 5 °C, and 12 °C, alongside *M. acetivorans* cultures grown at 2 °C and 37 °C. Green cells indicate presence, red cells absence. Notable shared compounds across environments include benzaldehyde, acetophenone, o-xylene, and pentanoic acid (Table 1). Overlap was highest at 200 cm and 12 °C, suggesting this thermal regime supports VOC profiles associated with acetoclastic methanogenesis. Unique *M. acetivorans* compounds (e.g., dimethyl sulfide, squalene, furfural) may serve as archaeal-specific biomarkers. These unique methanogen-associated VOC profiles underscore the potential of volatile signatures to trace archaeal activity and infer methanogenic pathways in permafrost-affected soils.

The overlap between BTL and *M. acetivorans* VOCs was most pronounced at 200 cm depth and 12 °C—conditions previously shown to favor acetoclastic methanogenesis—where 13 of the 16 compounds were shared with *M. acetivorans* grown at 37 °C, and 10 were shared with cultures grown at 2 °C. Several VOC classes were consistently detected across both systems. Aromatic compounds such as acetophenone, benzaldehyde, and o-xylene were ubiquitous, appearing in nearly all BTL incubations as well as both *M. acetivorans* temperature treatments. Long-chain alkanes and aldehydes, including tridecane, decanal, and dodecanal, were also present in both soil and culture samples, potentially reflecting shared processes such as lipid degradation or membrane remodeling. Phenol, detected in surface and 200 cm soil incubations and in the 2 °C *M. acetivorans* culture, may represent a byproduct of aromatic compound metabolism or microbial stress responses.

VOCs shared between *M. acetivorans* and BTL soil incubations were not limited to warm conditions. At 200 cm and 5 °C, ten overlapping compounds were identified, including butylated hydroxytoluene, benzaldehyde, and pentanoic acid, indicating that methanogenic VOC signatures persist under moderate cold stress. In contrast, compound overlap diminished substantially at −4 °C, where only six VOCs were shared between the BTL 200 cm incubations and either of the *M. acetivorans* culture temperatures. The greatest divergence occurred in the deepest BTL samples at 400 cm, where overlap with *M. acetivorans* VOCs was minimal. At this depth and −4 °C, only six shared compounds were detected, including benzaldehyde, butylated hydroxytoluene, and o-xylene, suggesting limited but persistent microbial metabolic activity under cryogenic conditions.

### Simulated methane production and lagged surface flux in warming thermokarst soils

**Figure 7:**
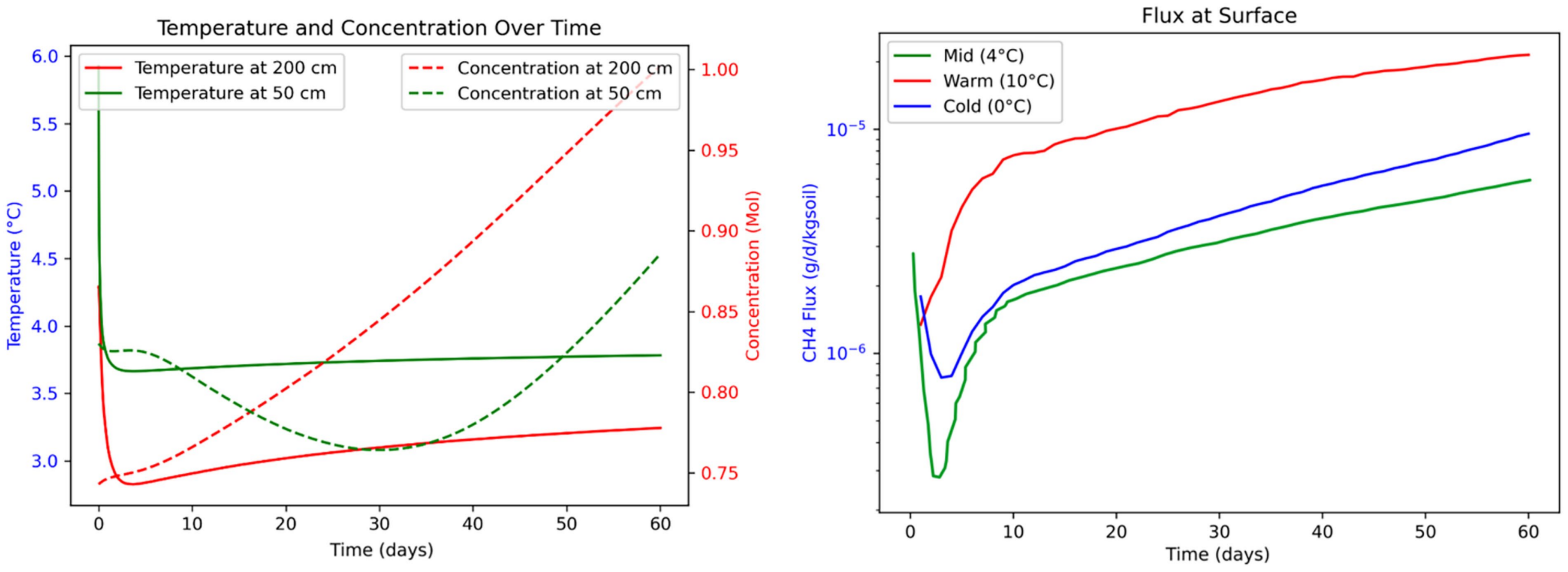
Numerical modeling of methane dynamics during 60-day mesoscale anaerobic incubations of thermokarst soils from Big Trail Lake. (Left panel) Simulated subsurface temperature (solid lines) and CH₄ concentration (dashed lines) at 50 cm (green) and 200 cm (red) depths. Temperatures reflect initial transient responses followed by stabilization, while CH₄ concentrations increase over time, with higher accumulation at 200 cm. (Right panel) Modeled CH₄ fluxes at the soil surface under three temperature regimes—Cold (0 °C, blue), Mid (4 °C, green), and Warm (10 °C, red)—highlight the exponential temperature sensitivity of CH₄ efflux. Warmer conditions lead to accelerated and sustained increases in surface CH₄ emissions, suggesting enhanced microbial methanogenesis and vertical transport. These simulations support empirical findings of temperature-amplified CH₄ release in thawing permafrost.

To evaluate whether observed CH₄ fluxes from warming thermokarst soils could be explained by coupled hydro-thermo-biogeochemical processes, we employed a 1D reactive transport model using a modified version of PFLOTRAN flow and reactive transport simulator. The model simulated CH₄ production, transport, and flux at the soil-atmosphere interface under different temperature boundary conditions over a 60-day summer period, following 130 days of spin-up to equilibrate thermal and hydrologic conditions (Supplementary Figures 6–10; Figure 7).

#### Methane production dynamics

Simulations under three temperature scenarios (0 °C, 2 °C, and 4 °C surface temperature) revealed distinct trends in CH₄ accumulation and vertical transport. Warmer temperature scenarios led to a more rapid thawing of permafrost and deeper active layers, which in turn would facilitate microbial methanogenesis at greater depths. This resulted in a time-dependent increase in CH₄ concentrations, particularly between 50 cm and 200 cm depths. Notably, in the 4 °C scenario, deeper zones (200 cm) began to accumulate higher CH₄ concentrations than shallower layers (50 cm) after approximately 15 days (Figure 7, right panel), suggesting that permafrost-proximal CH₄ production is initially limited by transport but accelerates as warming progresses.

#### Lag time between microbial activation and surface flux

A key insight from the model is the temporal lag between subsurface CH₄ production and its appearance at the ground surface. The 15-day delay observed in the 4 °C scenario highlights a temporal disconnect between microbial activity at depth and atmospheric CH₄ flux (Figure 7). This lag likely reflects physical transport constraints through saturated or partially frozen soils. The model also suggests that this lag is influenced by methane production rates and transport mechanisms, such as diffusion, ebullition, or advection (Elder et al., 2021).

#### Surface methane flux

Modeled CH₄ fluxes at the soil surface varied with warming intensity (Figure 7). Warmer scenarios led to more rapid and higher CH₄ emissions, peaking around day 45–50. The 4 °C case showed flux magnitudes of 1.0 × 10^−5^ g/day/kg soil, which is similar to those observed in our incubation experiments (Supplementary Figure 1), providing a strong validation of both the model and empirical measurements. In contrast, cooler scenarios exhibited more gradual flux increases, despite permafrost being closer to the surface, indicating that microbial CH₄ production is still governed by temperature-driven reaction kinetics rather than solely proximity to thaw front.

## Discussion

Permafrost thaw is a critical component of carbon flux dynamics, yet the microbial and chemical mechanisms driving CH₄ emissions remain poorly constrained in earth system models. In this study, we investigated how temperature affects subsurface microbial activity and volatile organic compound (VOC) emissions across a vertical profile of anaerobic permafrost soils within a thermokarst CH₄ hotspot in interior Alaska. Using short-term and long-term anaerobic incubations at three temperatures (−4 °C, 5 °C, 12 °C) and three depths (50, 200, 400 cm), we measured CH₄ fluxes, carbon isotopic composition (δ^13^-CH₄), VOC profiles, and microbial community dynamics using shotgun metagenomics. This multi-pronged approach allowed us to link biogeochemical processes with microbial functional potential across distinct thermal and depth gradients.

Our findings reveal that CH₄ emissions increased consistently with temperature, and isotopic enrichment (δ^13^-CH₄) suggests the concurrent influence of CH₄ oxidation, likely through anaerobic pathways such as iron-dependent methanotrophy. Metagenomic analysis revealed depth-stratified microbial communities that shifted compositionally with warming, including the activation of methanogens and iron-cycling methanotrophs in deeper soils. Correspondingly, VOC production was both depth- and temperature-sensitive, with distinct chemical fingerprints—particularly at 200 cm—coinciding with the emergence of metabolically active taxa such as *Methanosarcina*.

While numerical modeling indicates that CH₄ produced at depth may take at least 15 days to reach the surface—due to a combination of slow diffusive transport and microbial oxidation—VOCs may offer a more immediate signal of microbial metabolic activity. Although we did not measure VOCs and CH₄ simultaneously in the same experiment, separate laboratory incubations revealed that VOCs emerged during early stages of microbial activity, consistent with their role as byproducts of initial fermentation, stress response, or methanogenic transitions. VOCs are also subject to attenuation through sorption, biodegradation, or diffusion limits, but prior studies (Insam and Seewald, 2010; Peñuelas et al., 2014) suggest that they may diffuse more rapidly than CH₄ in some environmental contexts. Together, these results point to a thermally responsive subsurface biosphere that modulates CH₄ production and consumption. Although further work is needed to establish direct temporal relationships, the temperature-sensitive VOC profiles observed here support their potential as early chemical indicators of microbial activation and may help identify emerging CH₄ hotspots in permafrost-affected environments.

While numerical modeling indicates that CH₄ produced at depth may take at least 15 days to reach the surface, VOC signatures likely emerge earlier and independently of this physical delay. This suggests that VOCs reflect real-time microbial metabolic transitions rather than simply gas accumulation or transport. Taken together, these results point to a thermally responsive subsurface biosphere capable of modulating CH₄ flux through both production and oxidation pathways. Importantly, the distinct and temperature-sensitive VOC profiles offer promise as early chemical indicators of microbial activity, preceding detectable CH₄ flux and potentially serving as an early warning system for emerging CH₄ hotspots under continued Arctic warming.

### Depth-resolved microbial shifts and across the methane hotspot

Our investigation into microbial community dynamics across a vertical profile of anaerobic permafrost soil at a thermokarst hotspot reveals stratified and thermally responsive assemblages with implications for broader carbon cycling processes. At 50 cm, microbial communities were dominated by facultative anaerobes and metabolically flexible taxa, including *Pseudomonas*, *Arthrobacter*, and *Bradyrhizobium*, with relative abundances increasing at elevated temperatures. These taxa likely participate in organic degradation and nitrogen cycling, generating intermediates like acetate and lactate that fuel methanogenesis (Morris et al., 2013).

While methanogenesis is traditionally considered an obligately anaerobic process, recent findings have demonstrated the potential for methane production under oxic conditions in a range of ecosystems (Ernst et al., 2022; Günthel et al., 2019). Although our study focuses on anoxic soils, we acknowledge that oxic methanogenesis may also occur at the lake margin or within microoxic niches, and merits further investigation in future work. The prominence of *Pseudomonas*, a known degrader of complex carbon compounds, aligns with observations from active layer soils in Stordalen Mire, Sweden, where aerobic heterotrophs have been linked to enhanced carbon turnover during thaw (Hodgkins et al., 2014). Similarly, *Arthrobacter*—often associated with seasonal thaw layers in interior Alaska—reflects resilience in fluctuating redox and temperature regimes (Haan and Drown, 2021; Liu et al., 2023).

At 200 cm, the microbial community shifted toward increased abundance of *Bradyrhizobium* and detectable levels of *Methanosarcina*. At 400 cm—within historically stable, deep permafrost—warming induced a marked community shift, with *Ferrigenium* and *Sideroxydans* emerging in higher abundance. The activation of iron-oxidizing bacteria such as *Rhodoferax, Geobacter, Gallionella,* and *Sideroxydans* indicates that alternative electron acceptors like iron may play an underappreciated role in subsurface microbial metabolism, particularly under warming conditions (Li et al., 2024; Levar et al., 2017; Patzner et al., 2022; Berns-Herrboldt et al., 2025). These shifts suggest that microbial community structure in anaerobic permafrost is likely shaped by temperature as well as depth-related gradients in redox potential, moisture availability, substrate quality and *in situ* thermal conditions–all of which co-vary with depth and collectively influence microbial functional potential.

The substantial proportion of unclassified taxa—exceeding 50% relative abundance in some warm treatments at 400 cm—underscores the cryptic diversity of deep permafrost microbiomes. Similar patterns have been reported in metagenomic studies from thaw gradients in Alaska and Siberia, where a significant fraction of sequences remain unassignable to known lineages (Burkert et al., 2019; Woodcroft et al., 2018). Together, these findings emphasize the need for depth-resolved microbial assessments as a foundation for understanding permafrost carbon flux dynamics. The observed thermal responsiveness of microbial communities across depths sets the stage for exploring the functional strategies and ecological niches of key microbial groups driving carbon turnover in these dynamic environments.

### Methanogen distribution is cosmopolitan in permafrost hotspots but methanogenic potential is driven by microscale heterogeneity

#### Ecological niches of methanogenic hotspots

The methanogenic community composition observed in this study reflects specialized ecological strategies associated with CH₄ production in permafrost-affected soils, particularly within localized CH₄ hotspots. These zones—characterized by elevated CH₄ flux—are likely driven by microscale heterogeneities in redox gradients, substrate availability, and thaw-induced temperature shifts (Li et al., 2024; Lacroix et al., 2023; Knoblauch et al., 2018). Such environmental discontinuities create favorable anaerobic niches that support the proliferation of methanogens, the terminal agents of carbon mineralization in cold, anoxic systems (Schuur et al., 2015).

#### Dominant methanogenic pathways and taxa

**Figure 8:**
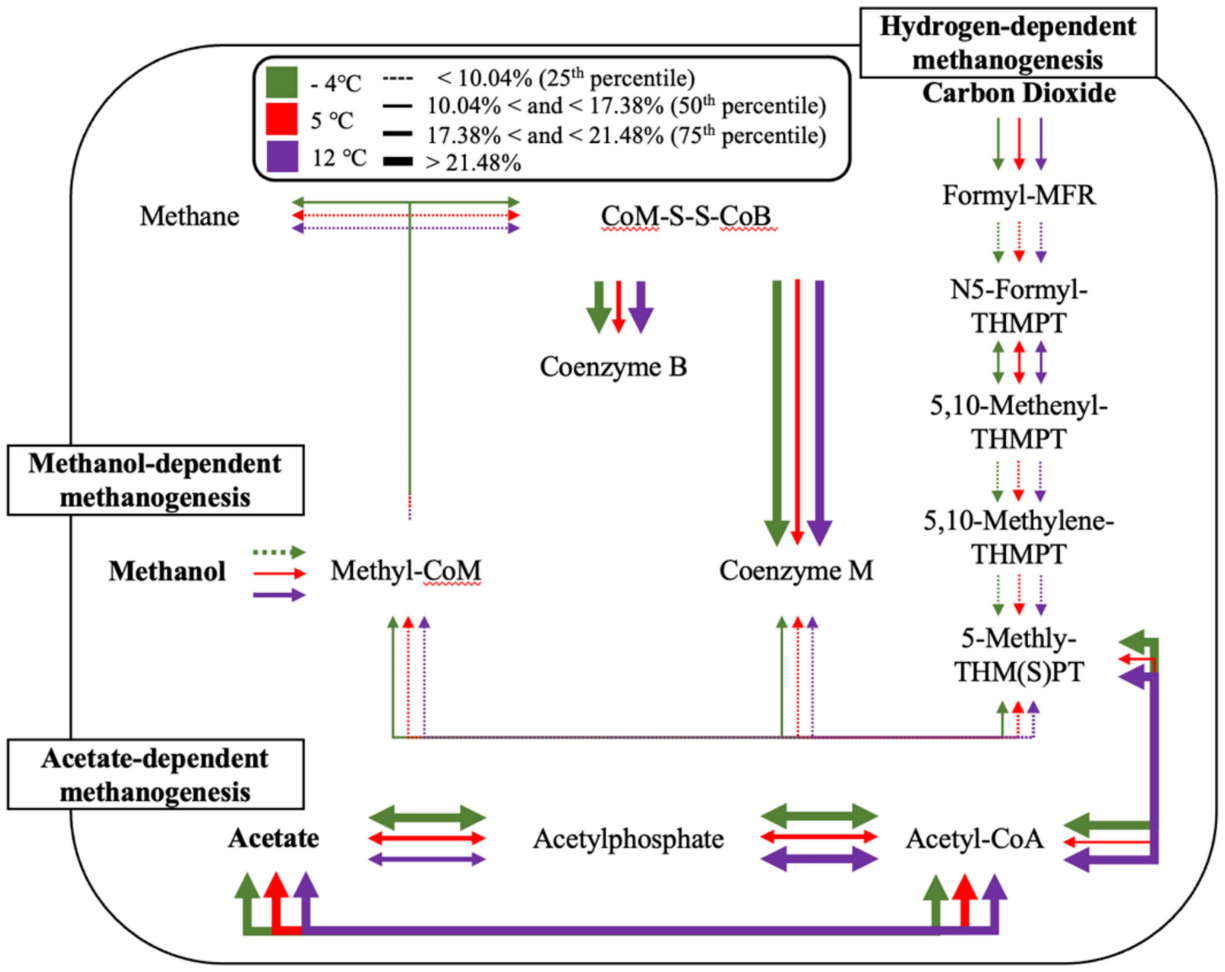
Temperature-dependent methanogenesis pathway activity inferred from metagenomic functional potential. Arrows indicate the relative abundance of key methanogenesis-associated genes across three temperatures: −4 °C (green), 5 °C (red), and 12 °C (purple). Line thickness corresponds to quartile ranges of relative abundance, with dotted lines representing <10.04% (25th percentile), thin solid lines between 10.04–17.38% (50th percentile), medium lines between 17.38–21.48% (75th percentile), and thick solid lines >21.48%. Pathways include hydrogenotrophic (right), acetoclastic (bottom), and methylotrophic (left) methanogenesis routes. Note: This diagram reflects the aggregate functional potential of the microbial community and does not imply that all pathways co-occur within a single organism. Gene presence was inferred from metagenomic annotations of unbinned, read-based data and should be interpreted as community-scale metabolic potential.

Across hotspot samples, members of the *Methanobacteriaceae* and *Methanomicrobiaceae* families were consistently present and abundant. Both represent hydrogenotrophic methanogens that utilize H₂ and CO₂ for CH₄ production (Figure 8; Supplementary Table 2). Their dominance implies that hydrogenotrophic methanogenesis is a key metabolic pathway in these zones, likely supported by upstream fermenters that generate molecular hydrogen under oxygen-limited conditions (Conrad, 2007; Conrad, 2020). This process is particularly favorable in permafrost environments where fermentation kinetics are slowed, allowing H₂ to accumulate to bioavailable levels (Wagner and Liebner, 2009).

The detection of *Methanosarcinaceae* across several hotspots is also notable, given their metabolic versatility (Ferguson and Mah, 1983). Capable of utilizing acetate, H₂/CO₂, and methylated substrates, these methanogens may be well suited to the transient and spatially variable substrate regimes associated with episodic thaw and detrital input (Coolen and Orsi, 2015). In contrast, *Methanotrichaceae* (formerly *Methanosaetaceae*), obligate acetoclastic methanogens, were detected more sporadically but showed elevated abundance in specific core depths. Their presence likely indicates longer-term anoxic zones where acetate is both available and stable, suggesting more mature, structured anaerobic microsites (Lacroix et al., 2023; Liu and Whitman, 2008; Steinberg and Regan, 2008).

Interestingly, methylotrophic methanogens such as *Methanomassiliicoccaceae* were also identified. These obligate H₂-dependent methylotrophs metabolize methylated compounds—substrates rarely emphasized in classical permafrost systems. Their presence suggests the in-situ production of methylated organics, potentially via the microbial degradation of osmolytes (e.g., glycine betaine) or peat-derived substrates (Borrel et al., 2013; Speth and Orphan, 2018). This highlights the importance of niche partitioning among methanogenic functional guilds and underscores the potential for overlooked pathways in CH₄ emissions.

In addition to methanogenic pathways, we queried the metagenomes for functional markers diagnostic of ANMEs, particularly those enriched in ANME-2 lineages (Supplementary Table 3) (Ouboter et al., 2024). These included multiheme c-type cytochromes (e.g., *mtrC*, *omcI*), subunits of membrane-bound hydrogenase complexes (*echABCDEF*), formate dehydrogenases (*fdhABCD*), and components of the Rnf complex (*rnfABCDEG*), which facilitate extracellular electron transfer and energy conservation. At 4 °C, we observed the highest cumulative abundance of these gene markers, suggesting a temperature-sensitive enrichment of ANME-related functional potential. Notably, several cytochrome and *fdh* genes showed marked increases between −4 °C and 4 °C, including *mtrC*, *omcI*, and *fdhA/B*, while *rnfC* and *rnfG* also increased. This pattern aligns with our isotopic observations of δ^13^C-enriched CH₄ under warming conditions and provides additional support for the involvement of AOM, potentially coupled to iron or other terminal electron acceptors (Ouboter et al., 2024; Haroon et al., 2013; Orphan et al., 2002). Although genome-resolved approaches were not performed, the gene-level signatures are consistent with AOM-associated taxa becoming more active or abundant with modest warming.

#### Vertical stability of methanogenic communities at 200 cm depth

Our metagenomic analysis further revealed that microbial community structure was primarily influenced by sediment depth, with temperature acting as a secondary factor (Supplementary Figure 3). Most strikingly, in our PCA the 200 cm samples formed a tightly clustered group across all incubation temperatures, in contrast to the broader dispersion observed at 50 cm and 400 cm. This pattern indicates not only a higher degree of community stability at intermediate depths, but also a remarkable resilience of methanogenic populations to temperature fluctuations.

Metagenomic evidence confirmed that the anaerobic microbial fraction at 200 cm is strongly dominated by methanogens. The consistent structure of these communities across thermal treatments suggests that methanogenesis at this depth may persist with minimal disruption under changing environmental conditions. This resilience could stem from stable redox profiles, sustained organic substrate availability, or long-term adaptation to cold anoxic niches—conditions conducive to robust methanogenic activity (Li et al., 2024; Cui et al., 2024).

This vertical stratification underscores the importance of depth-resolved functional assessments in thawing permafrost systems. The thermally buffered nature of methanogen-rich zones at 200 cm suggests that they may serve as persistent sources of CH₄, even under shifting temperature regimes. These findings point to a critical need to incorporate both functional guild dominance and vertical heterogeneity into CH₄ emissions models, particularly as permafrost regions undergo rapid ecosystem change.

### Microbial controls on methane emissions

The 60-day anaerobic incubation of 100 cm depth thermokarst soils from BTL also revealed a clear temperature dependence in CH₄ production and microbial functional potential. Methane mass and flux increased markedly with incubation temperature, with the highest accumulation observed at 10 °C, while flux plateaued under subzero conditions, particularly at −4 °C (Supplementary Figure 1). CO₂ production, in contrast, was relatively stable across temperatures following an initial spike during the first 7 days—likely due to residual oxygen—which subsided by day 15. After this transition to fully anoxic conditions, CO₂ production increased moderately at 4 °C and 10 °C but remained low at −4 °C and −30 °C.

These gas trends were mirrored in the metagenomic profiles, which showed strong temperature-linked increases in key methanogenesis genes. Specifically, the abundance of *methyl-coenzyme M reductase* (EC:2.8.4.1), *acetate kinase* (EC:2.7.2.1), and *formylmethanofuran dehydrogenase* (EC:1.2.99.5) rose consistently from −30 °C to 10 °C, reflecting enhanced hydrogenotrophic and acetoclastic methanogenesis with ecosystem warming (Supplementary Table 1). Methylotrophic pathways (e.g., *methanol co-methyltransferase*, EC:2.1.1.90) showed peak gene abundance at 4 °C, suggesting cold-adapted activity of these pathways at intermediate temperatures.

In contrast, aerobic CH₄ oxidation potential was minimal across all temperatures. *Methane monooxygenase* (EC:1.14.18.3), the canonical marker for aerobic methanotrophs, was detected at extremely low abundance throughout the incubation. Metagenomic data indicate that methanotrophic capacity is minimal under cold, anaerobic conditions, as shown by the consistently low abundance of methane monooxygenase genes across all temperatures. This suggests limited microbial CH₄ oxidation during winter. Combined with physical and process-based modeling, these findings support the hypothesis that CH₄ produced at depth may bypass oxidation and escape more readily through preferential pathways in frozen or partially thawed soils.

The temperature sensitivity of CH₄ production, quantified via Q₁₀ coefficients, further supports the role of microbial amplification with soil warming. Q₁₀ values reached ~14 by Day 22—well above typical ecosystem model values—indicating a sharp increase in CH₄ production per degree of soil warming (Figure 2). However, by Day 60, Q₁₀ values declined, a pattern not consistent with elevated CH₄ oxidation, given the genomic data. This decline may instead reflect substrate depletion, saturation effects, or temperature-induced shifts in methanogenic community composition.

Collectively, these findings suggest that soil warming leads to both elevated methanogenic potential and increased CH₄ flux in deep permafrost soils, while CH₄ oxidation remains largely inactive under anoxic, cold conditions. The resulting imbalance between CH₄ production and oxidation may contribute to greater CH₄ emissions during winter and early thaw periods—especially where structural thawing or preferential gas pathways allow CH₄ to escape before microbial mitigation can occur (Walter Anthony et al., 2024). In addition, thawing and rewetting events can induce mechanical disruption of ice-rich soil matrices, leading to the sudden release of CO₂ and CH₄ that had accumulated over prior time periods. These emissions may reflect past microbial activity rather than active metabolism at the time of thaw, complicating efforts to distinguish immediate biological responses from physical gas release. However, a key limitation of this 60-day incubation experiment is the potential accumulation of CH₄ in sealed microcosms, which may not capture the full range of diffusion, ebullition, or oxidation processes occurring under natural field conditions. The buildup of headspace CH₄ could artificially enhance local CH₄ concentrations, potentially triggering methanotroph activation that would otherwise be spatially restricted or redox-limited *in situ*. While CH₄ oxidation genes exhibited temperature- and CH₄-dependent increases, this result could partly reflect incubation artifacts, and the potential for transient or localized methanotrophic blooms cannot be excluded. Additional studies—both laboratory-based and field-deployed—are needed to constrain the feedbacks between CH₄ production, transport, and oxidation in geographically and geochemically diverse thermokarst environments.

### Depth-resolved VOCs as early indicators of subsurface microbial hotspots

The heatmap illustrates temperature- and depth-dependent patterns of VOC expression, revealing distinct chemical signatures across the permafrost soil profile (Figure 5). At 50 cm, warming to 12 °C induces a broad upregulation of VOCs, consistent with microbial activation and increased metabolic flux in the biologically active surface layer. Several VOCs remain elevated even after refreezing (−4 °C), suggesting persistence of volatiles or residual metabolic signals from prior thaw-induced activity. In contrast, the 200 cm depth—a more anaerobic and colder zone—exhibits a narrower but more diagnostic suite of VOCs, which become more pronounced under both warming and freezing conditions. These depth- and temperature-specific patterns point to microbial consortia, particularly methanogenic lineages such as *Methanosarcinales*, as being metabolically responsive to thermal perturbation. Their volatile byproducts likely reflect early stages of anaerobic carbon turnover. Given the low molecular weight and diffusivity of VOCs, their early appearance may precede detectable gas emissions and offer a window into real-time microbial activity. The distinct and consistent volatiles observed at 200 cm support the potential use of VOCs as biosensors of microbial reactivation, although concurrent CH₄ and VOC measurements in field settings will be essential for validation.

Organometallic compounds, benzenoids, and organic oxygen compounds were particularly enriched in high-temperature methanogen incubations (e.g., 200_12 °C), supporting active acetoclastic methanogenesis by *Methanosarcina* spp. These VOC classes have putative associations with cofactor biosynthesis, central metabolic intermediates, and increased biosynthetic activity during methanogenic growth (Nazaries et al., 2013; Thauer, 2011). In contrast, the elevated abundance of organic salts, organic nitrogen compounds, and phenylpropanoids under frozen or freeze–thaw conditions (e.g., 50 cm at −4 °C, 200 cm at −4 °C) likely reflects microbial turnover and cryo-adaptation processes, including protein denaturation, osmoprotectant accumulation, and nitrogen mineralization. While our monoculture studies inform potential links, broader community overlap and environmental complexity limit specificity. However, these VOC trends (Table 1) are consistent with findings from permafrost soils, where freeze–thaw cycling leads to nitrogenous compound accumulation and the release of stress-induced metabolites (Schuur et al., 2015; Mackelprang et al., 2011; Waldrop et al., 2023).

Benzenoids, lipid-like compounds, and hydrocarbons were consistently detected across depths and temperatures and may represent a core VOC group tied to both microbial maintenance and interspecies signaling. Especially when found in specific ratios or combinations, these metabolites may function as biosignatures of incipient methanogenic activity—prior to detectable CH₄ production. While it is true that CH₄ exhibits minimal sorption to soil components compared to many VOCs, the observed delay in surface CH₄ flux is more likely driven by slower diffusive transport through frozen or saturated soils and by microbial oxidation in the upper soil column. In contrast, VOCs—though more prone to sorption—are often produced rapidly during early microbial metabolic transitions. While we did not directly measure the temporal release of VOCs relative to CH₄ in this study, their detection in incubation headspace and supporting literature suggest they may emerge earlier under certain conditions, making them promising candidates for early microbial activity indicators. This early-warning capacity is particularly significant for CH₄ release forecasting, as it enhances our ability to detect and track hotspot emergence before CH₄ fluxes accelerate. Since VOCs likely diffuse more rapidly than CH₄ under partially thawed conditions and are readily detectable in incubation headspace, they represent a powerful, non-invasive tool for monitoring microbial biokinetics in thaw-sensitive landscapes. In other words, VOCs represent a novel or emerging diagnostic tool compared to traditional gas flux or genomic methods. Future work should aim to integrate these VOC fingerprints with metagenomic and transcriptomic datasets to strengthen causal links to methanogenic pathways. Field validation under natural thaw scenarios will be key for deploying VOC-based biosensors in boreal and Arctic observatories.

While our findings point to microbial origins for many VOCs, particularly those linked to acetoclastic methanogenesis, we acknowledge that VOC profiles in soil are shaped by both biological activity and the physicochemical properties of organic matter. Depth-resolved variation in VOC composition may reflect differences not only in microbial metabolism but also in the quality and quantity of organic carbon substrates. We have not yet fully disentangled the influence of organic matter composition on VOC production, but future work using advanced techniques such as Fourier Transform Ion Cyclotron Resonance Mass Spectrometry (FTICR-MS) or pyrolysis-GC/MS could help resolve the biotic and abiotic sources of key VOCs across thaw gradients.

### Integrating isotopic and functional data into permafrost feedback frameworks

#### Methane isotopes reveal uncertainties in thaw feedback models

Carbon isotopic signatures of CH₄ emitted from the BTL incubation experiments underscore important model gaps in how microbial CH₄ production responds to temperature. To constrain these emissions, we derived site-specific fractionation factors using the carbon isotopic composition of precursor organic matter and headspace CH₄ after 60 days of anaerobic incubation. We observed a temperature-dependent δ^13^C-CH₄ enrichment (−64.6‰ at −4 °C, −50.53‰ at 4 °C, and −38.90‰ at 10 °C), with corresponding α_tot_ values (1.042 to 1.014) that fall within or just outside the expected ranges for acetoclastic and hydrogenotrophic methanogenesis (Pellerin et al., 2022; Conrad, 2005). These shifts suggest changing methanogenic pathway dominance with warming.

A strong increase in *formylmethanofuran dehydrogenase* (EC:1.2.99.5), marker gene for hydrogenotrophic methanogenesis, relative to *acetate kinase* (EC:2.7.2.1), marker gene for acetoclastic methanogenesis, can be seen across increasing temperature, indicating a shifting preference toward hydrogenotrophic methanogenesis (Supplementary Table 1). This contrasts against results found in VOC incubations which show an increase toward acetoclastic production. The disparity can be explained with the duration of incubation, 60 days in CH₄ incubations against one-week periods in VOC incubations (Supplementary Table 2). Acetoclastic methanogenesis is energetically preferred to hydrogenotrophic methanogenesis yet shifts to hydrogenotrophic methanogenesis as a consequence of thaw is a common feature of permafrost thaw prior to the system returning to acetoclastic at the final stages of thaw (Heffernan et al., 2022; Liebner et al., 2015). Even so, the shift to hydrogenotrophic methanogenesis ultimately acts isotopically in the opposite direction as the isotope-temperature relationship derived in this study, ruling out production pathway shift as a likely cause. Instead, isotopic evidence points toward a methanotrophic enrichment of incubation headspace CH₄.

Contrasted against regional process-based model estimates, CH₄ isotopic emissions such as those from Oh et al. (2022) suggest δ^13^C-CH₄ values near −69 ± 6‰, which aligns more closely with our cold-incubation (−4 °C) headspace values but diverges significantly at warmer temperatures (Oh et al., 2022). This discrepancy highlights an urgent need for site-specific isotope-enabled modeling approaches in thermokarst landscapes (Treat et al., 2015). Additionally, our measured Q₁₀ of 7.9 exceeds values embedded in many Earth system models [typically ~4.4; Pumpanen et al., (2008)] yet within the large range of Q_10_ observed in permafrost ecosystems [1.2–22, Heslop et al. (2020)], signaling that current representations of temperature sensitivity may substantially underestimate emissions in warming permafrost.

#### VOCs as predictive biosignatures

Our numerical modeling results provide a mechanistic framework to estimate the timing and magnitude of CH₄ emergence at the surface under warming conditions. The model predicts a ~ 15-day lag between initial CH₄ production at depth and its surface expression in the 4 °C scenario, highlighting a temporal disconnect between subsurface microbial activity and atmospheric CH₄ flux. Although VOCs were not explicitly included in the model and were not temporally co-measured with CH₄ flux, their detection in endpoint incubations—particularly in treatments with elevated microbial activity—suggests that VOCs may arise earlier in the microbial activation sequence. When considered alongside the model’s prediction of delayed CH₄ emergence, this pattern supports the hypothesis that VOCs may serve as earlier indicators of microbial activity preceding CH₄ flux. VOCs—produced during initial stages of microbial metabolism, particularly during transitions from aerobic to anaerobic respiration—are more volatile and diffusible (Ghirardo et al., 2020; Insam and Seewald, 2010; Peñuelas et al., 2014). The model’s prediction of increased methanogenesis near thaw fronts, coupled with observed VOC biomarkers (e.g., methylated sulfur compounds and low-molecular-weight alcohols), suggests that VOCs may offer a real-time glimpse into microbial activation zones, even before CH₄ accumulates sufficiently to breach the surface. This reinforces the value of VOC monitoring as a predictive tool for CH₄ hotspots in thawing permafrost environments and highlights the need to integrate microbial early-warning signatures into Earth system models.

#### Iron-AOM as a suppressed but critical methane sink

Pellerin et al. (2022) found high Fe^2+^ in lake cores at BTL, indicating iron reduction, and was unable to fully connect it to other oxidation targets like acetate (Pellerin et al., 2022). Although isotopic enrichment in δ^13^CH₄ is consistent with CH₄ oxidation, we did not detect strong functional or taxonomic signals for canonical AOM pathways. Our hypothesis that Fe-AOM contributes to methane attenuation is based in part on previous geochemical analyses at Big Trail Lake, which identified elevated concentrations of ferrous iron (Fe^2+^) and minimal levels of alternative electron acceptors such as sulfate and nitrate (Pellerin et al., 2022). While our cores were collected from the lake margin and not co-located with those of Pellerin et al., the general trend of low sulfate supports our inference that sulfate-dependent AOM (S-AOM) is likely limited at this site. Functional gene annotations targeting canonical sulfate reduction pathways were also rare in our metagenomic data, further suggesting that microbial methane oxidation may be coupled to iron or other non-sulfate electron acceptors under anoxic conditions.

The observed enrichment could also reflect partial oxidation via non-canonical mechanisms such as iron-mediated AOM, likely coupled to iron reduction for methanotrophy, which may be a significant but underrepresented sink at BTL. A marked increase in the methane monooxygenase gene (sMMO) with warming, together with a concurrent rise in *Rhodoferax* and *Geobacter* (Supplementary Figure 4), known iron-reducers, points to temperature-sensitive microbial iron cycling (Ettwig et al., 2016; Weber et al., 2006). While our incubations were strictly anaerobic, these signals support the presence of iron-mediated methanotrophic CH₄ oxidation—a process demonstrated to significantly reduce CH₄ flux in sediments (Guerrero-Cruz et al., 2021; Bar-Or et al., 2017).

However, AOM is rarely parameterized in large-scale carbon models, despite strong evidence that microbial iron reduction co-occurs with methanotrophy in thawing soils (Pellerin et al., 2022; Tveit et al., 2019). The absence of an AOM term, particularly one coupled to redox-active metals, likely biases flux predictions high and omits key feedback regulation mechanisms. Incorporating Fe-AOM pathways and other alternative electron acceptor pathways could significantly revise the net CH₄ balance within the subsoil of rapidly thawing systems.

#### Toward isotope- and VOC-enabled monitoring frameworks

In addition to isotopic data, our work suggests that VOCs offer a tractable proxy for *in situ* microbial processes and may serve as real-time biosensors for thaw progression. Specific compound classes (e.g., benzenoids, phenylpropanoids, and hydrocarbons) were correlated with distinct microbial functions and thermal states, potentially offering signatures of methanogenesis, oxidative stress, or lipid turnover (Mackelprang et al., 2011; Boone et al., 2025).

These VOC fingerprints, if integrated into remote sensing or flux chamber monitoring strategies, could complement CH₄ isotope data to triangulate the origin, pathway, and oxidation history of CH₄ emissions. Incorporating VOCs into Earth system models would allow for dynamic coupling between biotic function and gaseous emissions, especially as biosensor technology matures (Zhou et al., 2024; Hess-Dunlop et al., 2025). Coupling these tools with functional genomic markers—such as those identified in this study and our recent report (Smallwood et al., 2025)—can advance monitoring frameworks beyond static carbon inventories, enabling dynamic, process-informed forecasting feedback models that are responsive to abrupt thaw scenarios (Schuur et al., 2015; Lenton et al., 2024).

## Conclusion

Permafrost thaw is accelerating Arctic CH₄ emissions, but the dynamics of microbial activation and gas transport remain undercharacterized—especially in abrupt thaw features like thermokarst lakes (Turetsky et al., 2020; Walter Anthony et al., 2018; Walter Anthony et al., 2021). In this study, we examined a CH₄ hotspot at Big Trail Lake using temperature-gradient anaerobic incubations, VOC profiling, stable isotope tracing, and metagenomics.

We found that CH₄ production increased nonlinearly with warming, and isotopic signatures indicated active anaerobic oxidation of methane (AOM), likely coupled to iron reduction. While methanogenic community structure remained relatively stable, the presence of methane oxidation genes and temperature-sensitive isotope enrichment suggested functional shifts not detectable by taxonomy alone.

Depth-resolved VOC profiles showed distinct chemical fingerprints of microbial activity, especially at 50 and 200 cm, with signatures resembling those from pure *M. acetivorans* cultures. These compounds emerged well before detectable CH₄ surface fluxes, supporting VOCs as early indicators of microbial biokinetics. Complementary modeling showed that subsurface CH₄ production led to surface emission lags of up to 15 days, while VOCs—while only measured during 7-day incubations—likely diffused more rapidly, reinforcing their utility as lead indicators (Ghirardo et al., 2020; Insam and Seewald, 2010; Peñuelas et al., 2014).

Our integration of experiments and reactive transport modeling highlights the importance of combining microbial, chemical, and physical data streams to understand microbial CH₄ dynamics. Future work should explore genome-resolved metagenomics, metatranscriptomics, and field-scale VOC sensing to improve detection of early biogeochemical transitions and support model calibration under changing Arctic conditions.

## Data Availability

Shotgun metagenomic sequencing data are available in NCBI under BioProject accession no. PRJNA1330893 (for biosample accession numbers, see Supplementary Data Sheet 2).
