# Supplementary material for: Microbial and chemical predictors of methane release from a stratified thermokarst permafrost hotspot
Source: Front Microbiol. 2025 Oct 10;16:1657143. doi: 10.3389/fmicb.2025.1657143 (PMC12549645; doi:10.3389/fmicb.2025.1657143)
Supplement: Supplementary file 1 [file Data_Sheet_1.docx]

**Availability of data**

TILES analysis of untargeted volatilomics using 2D-GC-MS quantification can be found in Supplementary Data Sheet 1. Shotgun metagenomic sequencing data are available in NCBI under BioProject accession no. PRJNA1330893 (for biosample accession numbers, see Supplementary Data Sheet 2).

**Supplemental Figures**


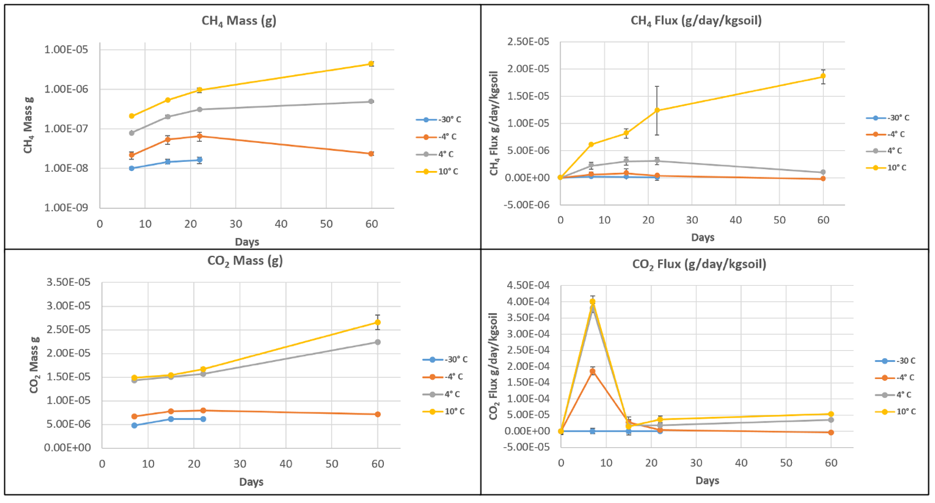


Supplemental Figure 1 Temporal trends in methane (CH₄) and carbon dioxide (CO₂) production and flux from anaerobic mesocosm incubations of Big Trail Lake (BTL) soil at four temperatures: -4°C, 0°C, 5°C, and 12°C. Top panels: CH₄ mass (left) and CH₄ flux (right) over 60 days, showing highest methane production and emission rates at 12°C, indicative of enhanced methanogenic activity under warmer conditions. Bottom panels: CO₂ mass (left) and CO₂ flux (right), with a sharp initial spike in CO₂ flux at 12°C followed by stabilization, reflecting early-stage microbial respiration and organic carbon turnover. Data demonstrate temperature-dependent microbial activation and carbon mineralization, with implications for greenhouse gas release from thawing permafrost.


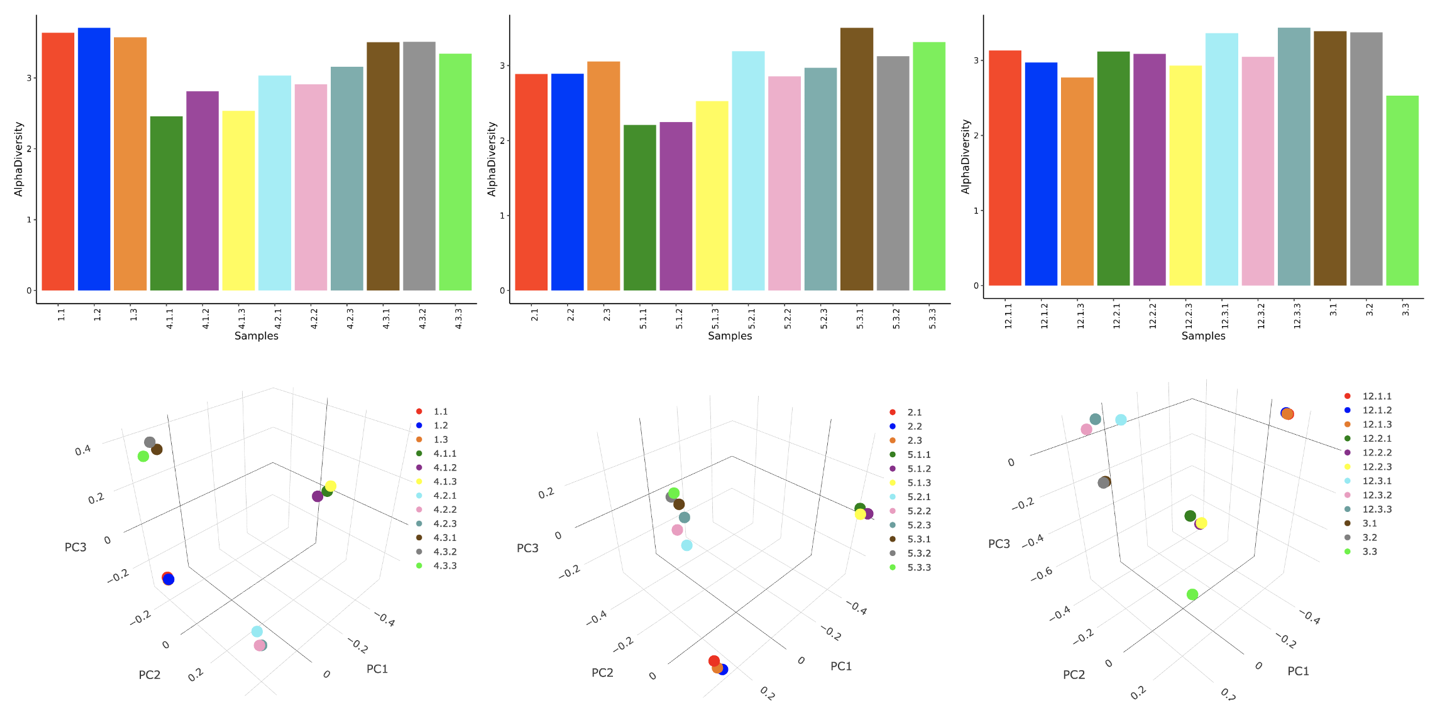


Supplemental Figure 2 Alpha diversity and microbial community structure across thermokarst soil incubation samples from Big Trail Lake. (Top panels) Bar plots show alpha diversity (e.g., Shannon index) for each biological replicate across soil depths (50 cm, 200 cm, 400 cm) and incubation temperatures (−20 °C, −4 °C, 5 °C, and 12 °C). (Bottom panels) 3D Principal Coordinates Analysis (PCoA) plots based on Bray-Curtis dissimilarity illustrate microbial community composition across the same samples, with clustering reflecting depth and thermal treatment. Sample labels correspond to depth, temperature, and biological replicate as follows: 1.1–1.3: 50 cm, −20 °C (R1–R3); 2.1–2.3: 200 cm, −20 °C (R1–R3); 3.1–3.3: 400 cm, −20 °C (R1–R3); 4.1.1–4.1.3: 50 cm, −4 °C (R1–R3); 4.2.1–4.2.3: 200 cm, −4 °C (R1–R3); 4.3.1–4.3.3: 400 cm, −4 °C (R1–R3); 5.1.1–5.1.3: 50 cm, 5 °C (R1–R3); 5.2.1–5.2.3: 200 cm, 5 °C (R1–R3); 5.3.1–5.3.3: 400 cm, 5 °C (R1–R3); 12.1.1–12.1.3: 50 cm, 12 °C (R1–R3); 12.2.1–12.2.3: 200 cm, 12 °C (R1–R3); 12.3.1–12.3.3: 400 cm, 12 °C (R1–R3).


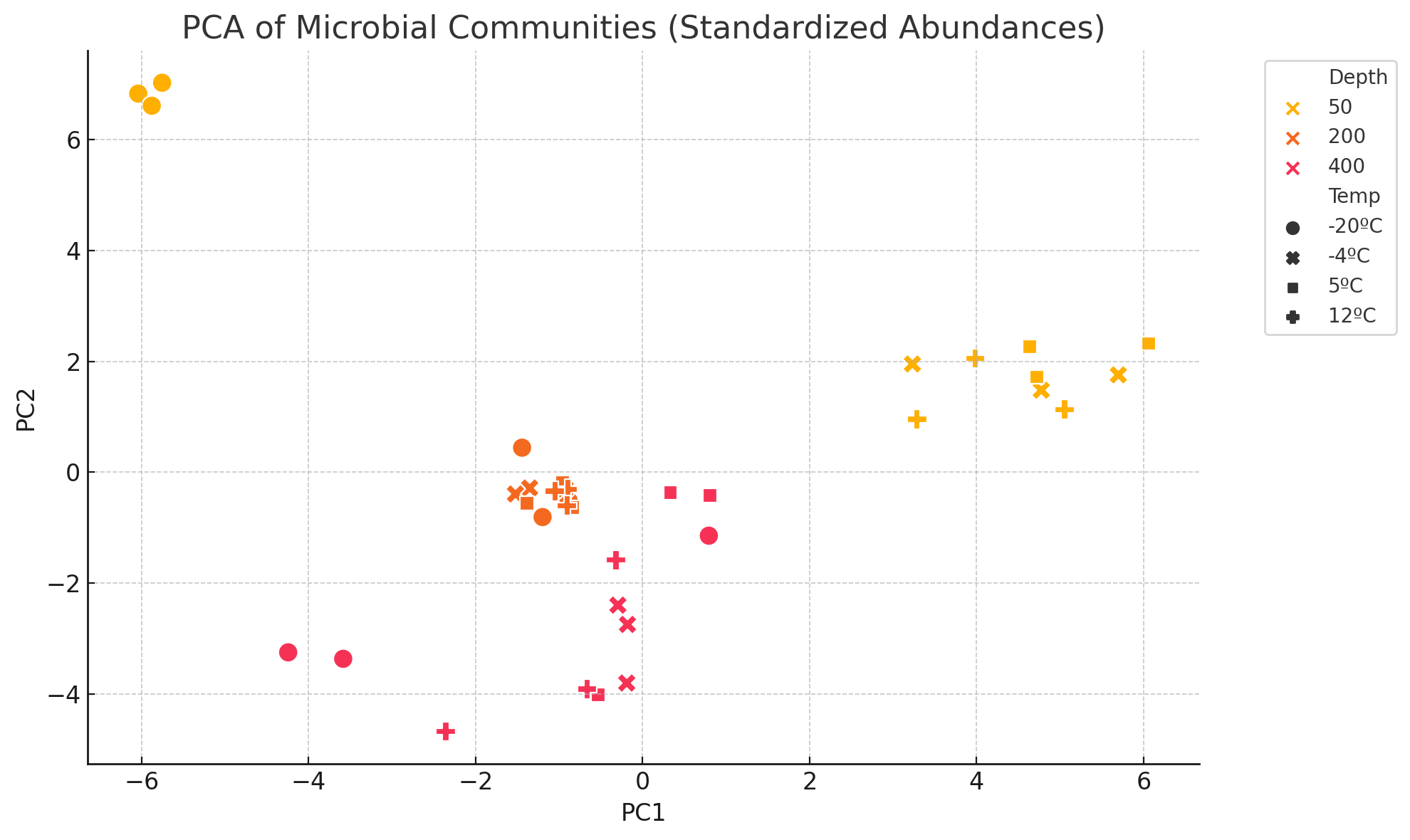


Supplemental Figure 3 **Principal Component Analysis (PCA) of Microbial Community Composition Across Depth and Temperature Gradients.** This PCA plot visualizes the standardized relative abundances of microbial genera across sediment samples varying by depth (50 cm, 200 cm, 400 cm) and incubation temperature (−20ºC to 12ºC). Each point represents a sample, colored by depth and shaped by temperature. Clustering patterns indicate that both depth and temperature influence community structure, suggesting environmental selection pressures on microbial assemblages.

Supplemental Table 1 Relative abundance of metagenomic genes associated with methane-cycling processes across core depths and incubation temperatures. The heatmap displays normalized read counts for key genes involved in methanogenesis and methane oxidation across permafrost core depths (50 cm, 200 cm, and 400 cm) incubated at −4°C, 5°C, and 12°C. Warmer colors (red) indicate higher relative abundance, while cooler colors (blue) indicate lower abundance.


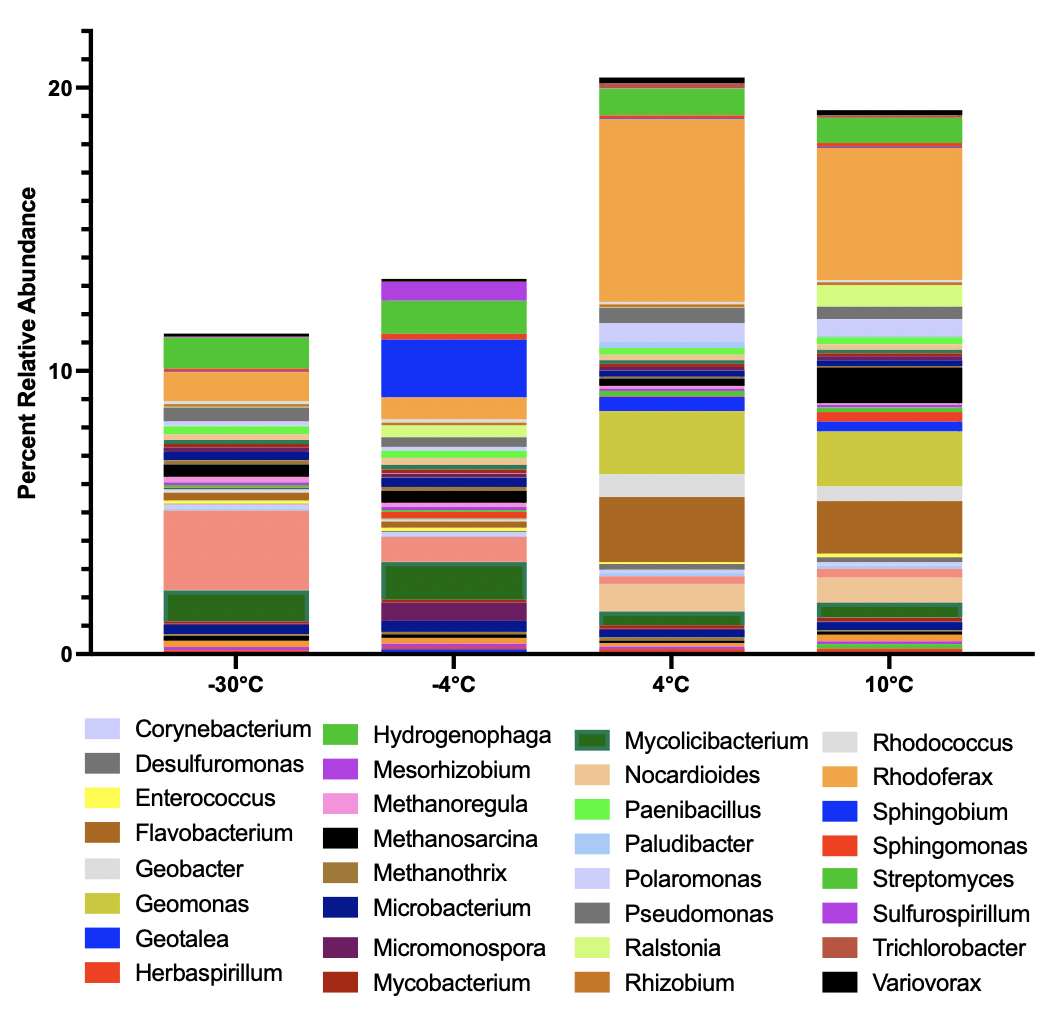


Supplemental Figure 4 Relative abundance of bacterial and archaeal genera across four temperature treatments (–30 °C, –4 °C, 4 °C, and 10 °C) during anaerobic incubations of permafrost-derived soils. Warmer temperatures (4 °C and 10 °C) resulted in higher total relative abundance and compositional shifts, including strong enrichment of *Rhodoferax*, *Geomonas*, and *Rhizobium*. These shifts suggest activation of facultative anaerobes and metal-reducing taxa under moderate thermal input. In contrast, colder incubations (–30 °C and –4 °C) displayed reduced diversity and distinct community composition. Genera involved in methane cycling—*Methanoregula*, *Methanosarcina*, and *Methanothrix*—also varied with temperature, indicating a thermally responsive microbial network relevant to subsurface carbon transformations and methane release during permafrost thaw.

Supplemental Table 2 Temperature-dependent detection of enzyme gene relative abundance involved in methane-related metabolism including methanogenesis-associated and methane-oxidation pathways in Big Trail Lake (BTL) thermokarst metagenomes. Enzymes annotated through KEGG were grouped by pathway (e.g., glycolysis/gluconeogenesis, pyruvate metabolism, methane metabolism), with corresponding KEGG identifiers and reaction numbers. Detection across incubation temperatures (–30°C, –4°C, 4°C, 10°C) is indicated by filled cells. Enzymes in methane metabolism (e.g., MCR, MTD, FTR) appeared predominantly at 4°C and 10°C, suggesting a temperature threshold for archaeal methanogenic activity.

Supplemental Table 3 Abundance of functional marker genes associated with anaerobic methane-oxidizing archaea (ANMEs) across incubation temperatures. The left table shows raw hit counts of open reading frames (ORFs) annotated to multiheme c-type cytochromes, hydrogenase complexes (ech genes), formate dehydrogenases (*fdh* genes), and components of the Rnf complex—all commonly enriched in ANME-2 archaea. The right table reports the normalized abundance of each gene, scaled to the total number of sequences assigned to functional annotations per sample (noted at bottom). Peak normalized abundance of ANME markers was observed at 4°C, including *mtrC*, *omcI*, *fdhA*, and *rnfC*, suggesting modest warming enriches the potential for anaerobic methane oxidation (AOM). These findings support functional evidence for AOM activity and complement isotopic observations reported in the main text.

Supplemental Figure 5 Comparative presence–absence matrix of volatile organic compounds (VOCs) identified in Methanosarcina acetivorans C2A (MaC2A) pure cultures and Big Trail Lake (BTL) thermokarst soil incubations across depth and temperature gradients. Compounds were annotated via KEGG and grouped based on occurrence: (i) VOCs overlapping between MaC2A cultures and BTL incubations at 200 cm depth (top section), (ii) VOCs found in BTL incubations at 200 cm but not in MaC2A (middle section), and (iii) compounds exclusive to MaC2A cultures (bottom section).


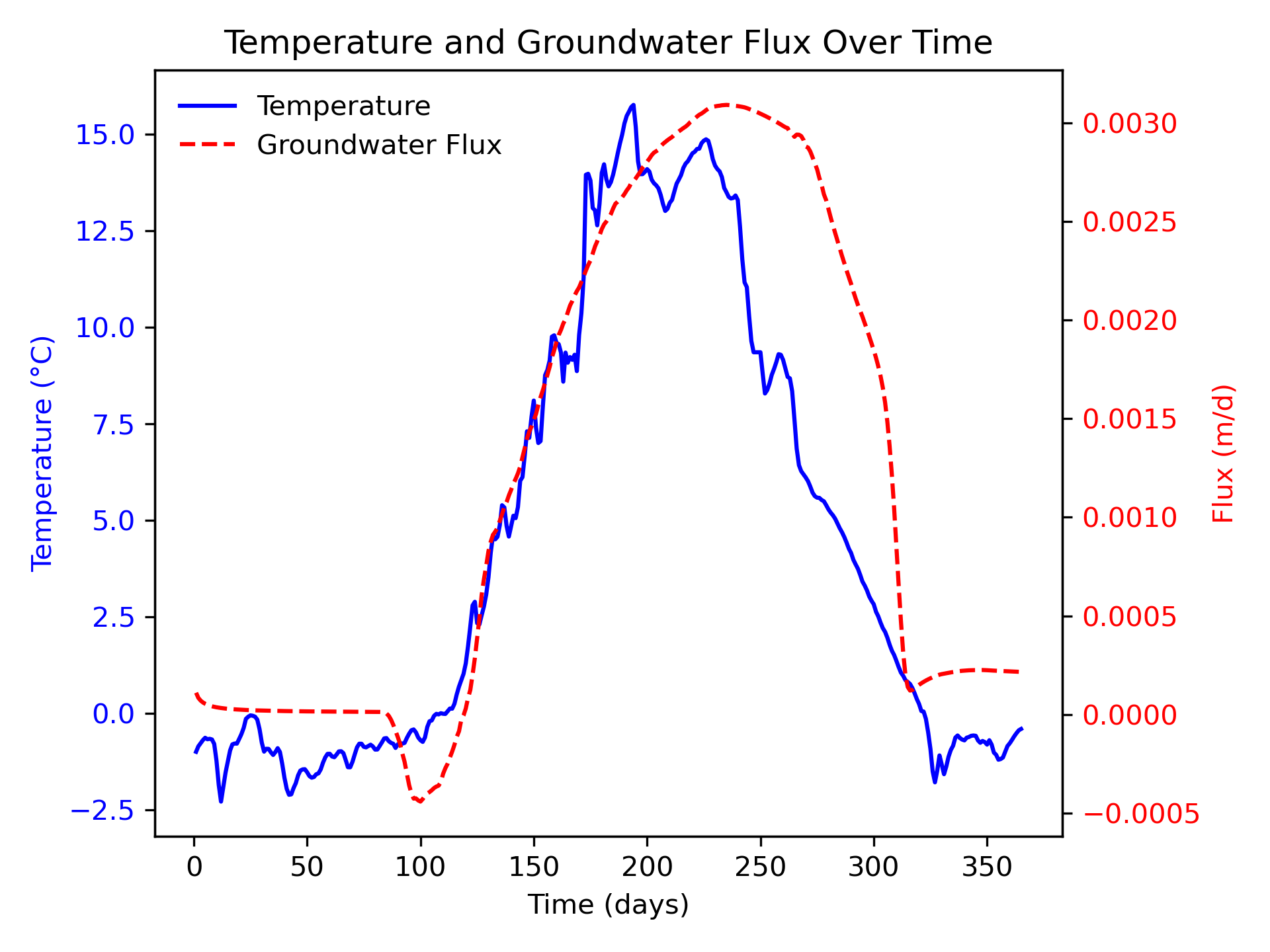


Supplemental Figure 6 Boundary conditions for 1D PFLOTRAN models. The top boundary condition is surface temperature data from the NOAA-CIRES 20th Century Reanalysis (V2) dataset (Comp et al., 2011). The bottom boundary condition is a liquid flux based on groundwater flow relative to land cover type (i.e. thermokarst lake – Walter Anthony et al. 2024).


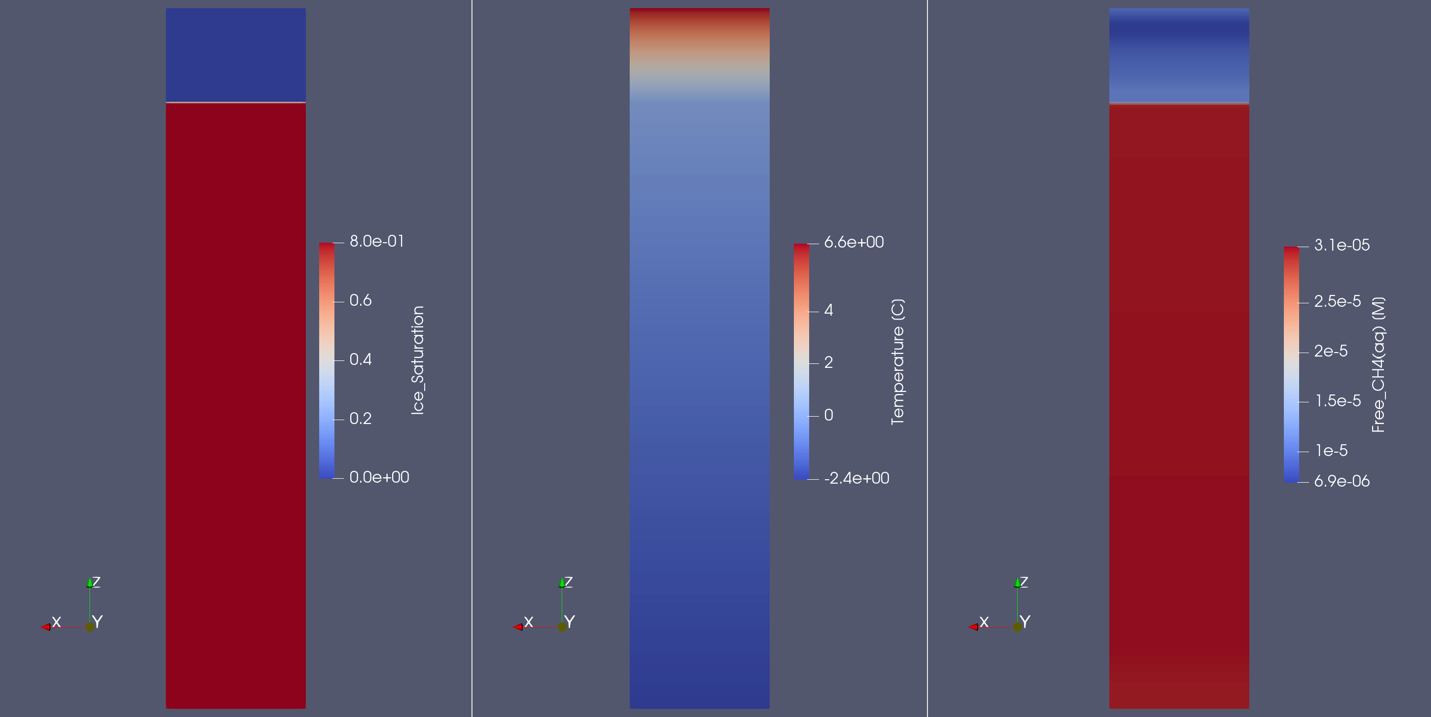


Supplemental Figure 7 Initial conditions after 130 days of simulation showing ice saturation, temperature, and CH_4_ concentration. The domain is (1 m × 1 m × 5 m) with one-dimensional discretization in the vertical direction at 1 cm resolution.

Supplemental Figure 8 Comparison of two different temperature scenarios. TOP – Normal temperature conditions where surface temperature boundary condition is set to 4ºC and simulated for 60 days. Bottom – Cold scenario where surface temperature is 0ºC.


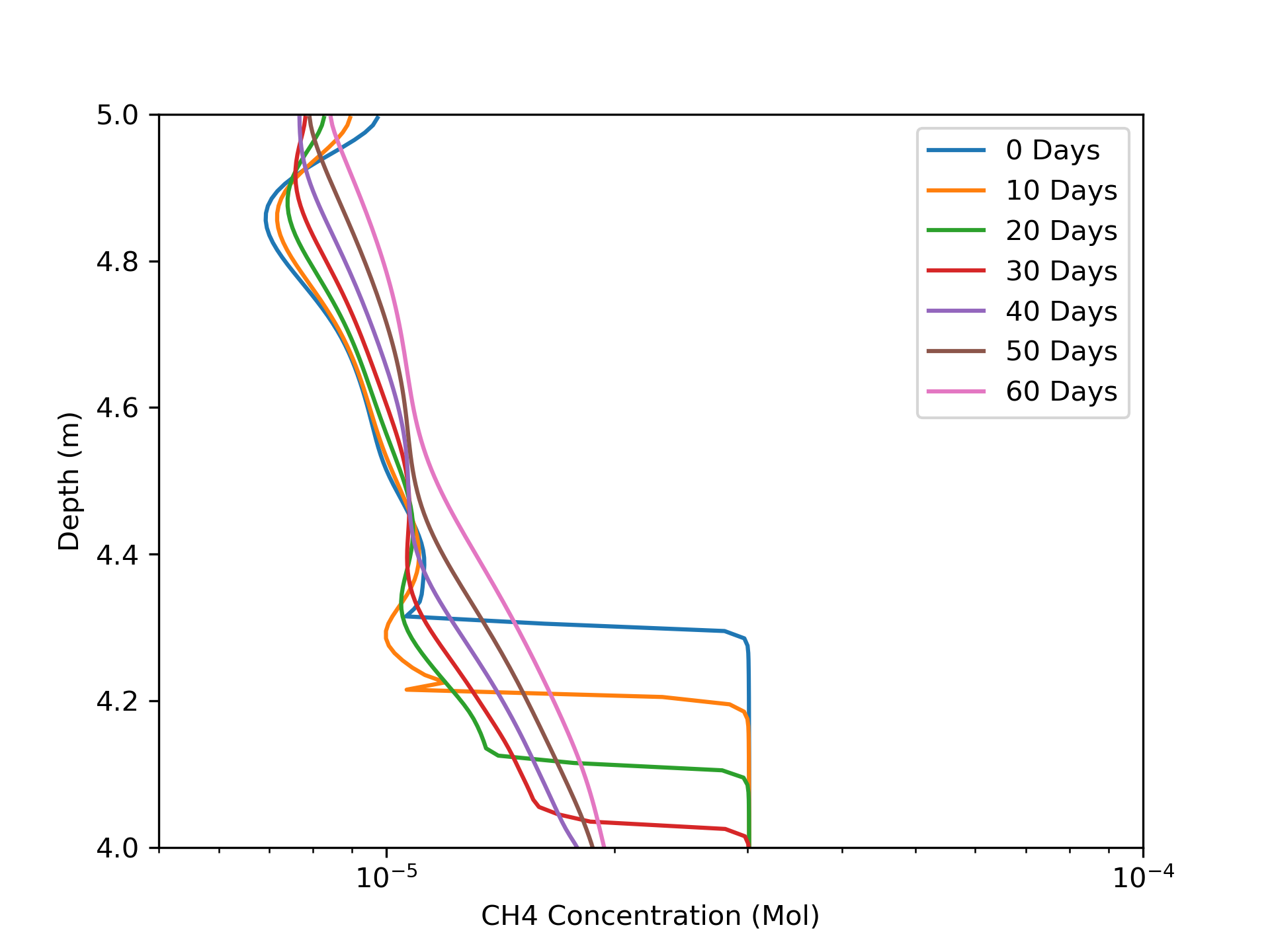


Supplemental Figure 9 Vertical profiles of methane concentration (mol) over a 60-day simulation at 4 °C, representing a moderate warming scenario. CH₄ concentrations are plotted as a function of depth from 5.0 m to 4.0 m. Over time, methane accumulates at shallower depths (near 4.2–4.4 m), reflecting upward diffusion from deeper production zones as permafrost thaws. Early time points (Days 0–30) show similar surface CH₄ concentrations, while later time points (Days 40–60) exhibit a marked increase in methane near the surface, indicating enhanced production and transport. The progressive deepening of the thaw front under warming enables this methane mobilization from depth to shallower layers.

Methane concentration as function of depth for 0ºC


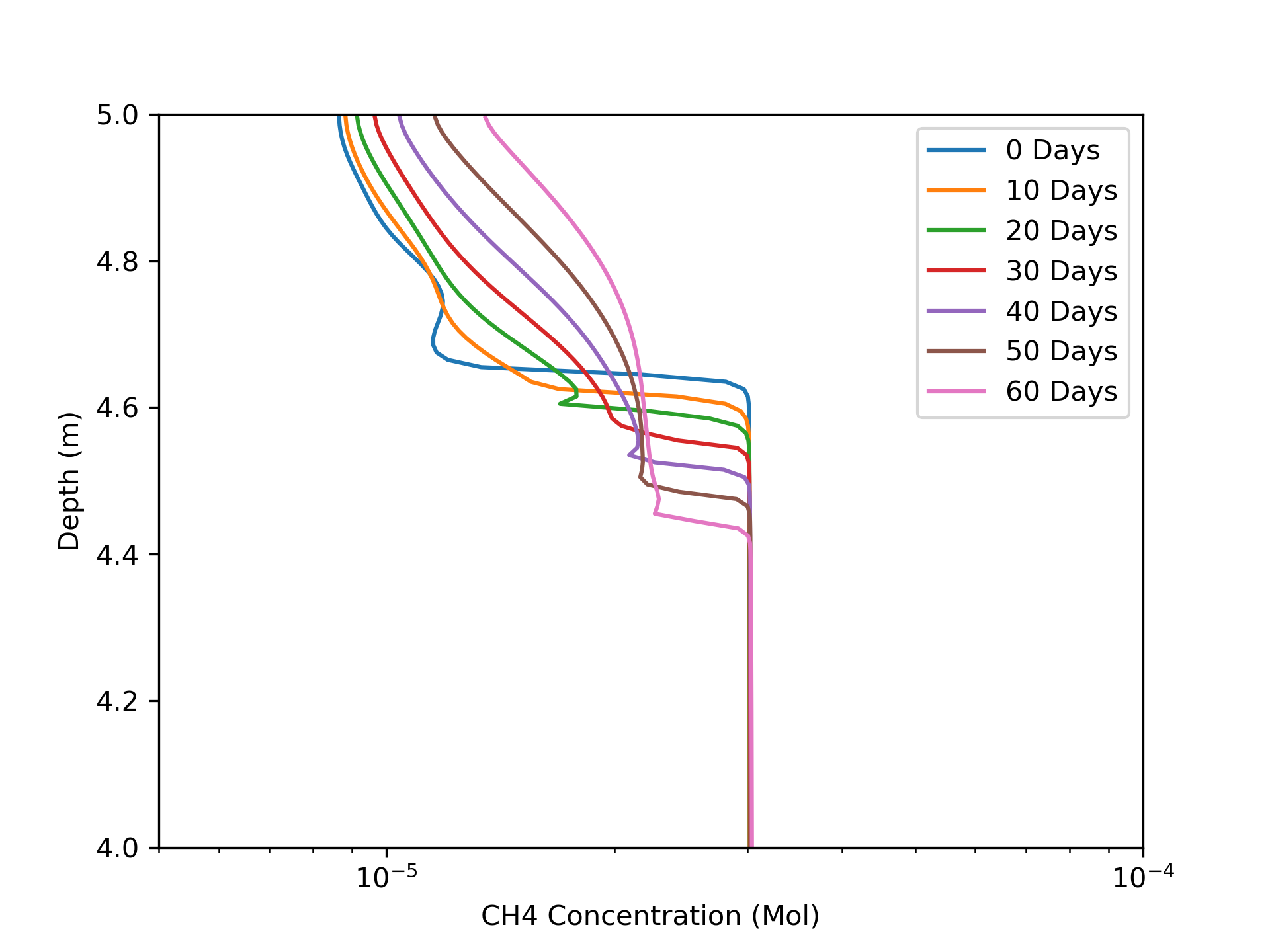


Supplemental Figure 10 Methane concentration as a function of depth for the 0ºC temperature scenario. Note that methane concentration at the surface increases consistently over the 60-day simulation.
